# Nonequilibrium Theory for Molecular Machine Design

**Published:** 2026-05-09

**Authors:** Ying-Jen Yang, Ken A. Dill

**Affiliations:** Laufer Center of Physical and Quantitative Biology, Stony Brook University; Laufer Center of Physical and Quantitative Biology, Stony Brook University; Department of Physics and Astronomy, Stony Brook University and Department of Chemistry, Stony Brook University

## Abstract

Modeling the dynamical flows on networks of biomolecular machines often entails computing node populations and edge fluxes with Master Equations and correlating machine performance with entropy production. But this alone is not sufficient for design, optimization and evolution because it doesn’t treat cost-benefit tradeoffs, or small-system misflows (backsteps, futile cycles, ineffective actions), or differential properties for flow design. Here we develop CFT Design, based on the recently developed Caliber Force Theory (CFT). We apply it to: designing faster molecular motors through “traffic control”; optimizing speed, energy, and accuracy in kinetic proofreaders; and designing better enzyme inhibitors. CFT Design provides a general framework for optimizing nonequilibrium flow networks.

## NETWORK FLOWS NEED DESIGN PRINCIPLES.

We are interested in network flows—fluids, particles, energies; trafficking of vehicles or goods; ecological movements of organisms and species; and especially the cyclic processes of biomolecular machines. These dynamics are commonly modeled by stochastic dynamical models where a traveling agent “rolls a die” to determine when and where to transition. For simple models of biomolecular machines, this die roll comes from the thermal noise of climbing energy barriers, described by Arrhenius-type exponential waiting times. Today’s modeling computes probability distributions and edge flows using Master Equations or Markov State Models, often focusing on correlating machine performance with entropy production [1–9].

What is missing for optimizing and designing flow networks? First, we need a way to treat the costs and benefits of flows. Whereas equilibrium (EQ) thermodynamics often balances the tradeoffs of heat and work, for example in Carnot cycles, network flow streams can have a much broader range of desirability properties. Product supply chains have dollar costs and benefits; biochemical pathways produce valued biomolecules and waste products; biomolecular proofreading trades off speed, accuracy and energy costs; etc. And, networks have time costs, which depend not only on how fast an agent takes a local transition, but also on slowdowns due to wrong turns, futile cycles, or dead ends. Network flow design theory must handle these more global *topological misflows* [10–12].

Second, non-equilibrium (NEQ) physics has not had the same level of powerful mathematical relationships that EQ has had, for example, establishing analytical relationships that connect flow variables to forces and constraints across different networks. This contrasts with EQ thermodynamics mathematics which relates complete variable sets, such as (U,V,N), to their conjugate forces (T,p,μ) and to whatever are the constraints that are imposed upon the system in the problem at hand. Here, we develop principles for the design, optimization, and evolution of flows under constraints. Based on the recent Caliber Force Theory (CFT) [13, 14], we establish the observable-force conjugacy for NEQ dynamics to formalize a framework of flow design. We apply the framework to three biomolecular machines with distinct types of misflows: correcting backstepping in the F_1_-ATPase motor [15]; optimizing branching across incorporation or correction cycles in kinetic proofreaders [16–19]; and strengthening dead-ends or futile cycles in enzyme inhibition [20–22].

## CALIBER FORCES GIVE FLOW DESIGN RULES.

**FIG. 1.**
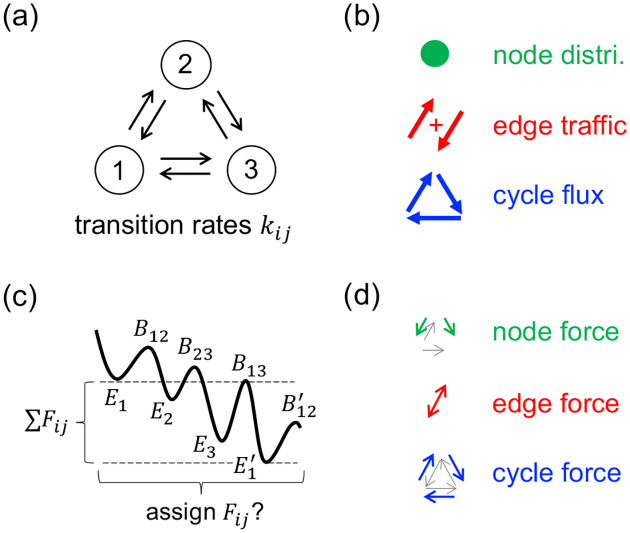
A simple 3-state machine process, with CFT properties defined. (a) A Markov network. (b) The observables: node probability distributions, symmetric edge traffics, and directed cycle fluxes. (c) Commonly modeled using an Arrhenius energy landscape $\left(E_{n},B_{ij}\right)$ and a cyclic drive $\left(\sum_{(ij)\in c}F_{ij}\right)$ that tilts the landscape. The primed variables are the unprimed shifted by $\sum F_{ij}$. There is a gauge ambiguity in distributing the drive onto each edge—many combinations of $F_{ij}$ and $E_{n}$ lead to the same dynamics. (d) CFT resolves this ambiguity by deriving conjugate forces as affinities to node dwelling, edge exchange, and cycle completion.

To begin, we first summarize the relevant parts of Caliber Force Theory (CFT), a principled general treatment of non-equilibria. Consider a molecular machine modeled as a Markov jump process on a fixed network state space (e.g., Fig. 1a). As illustrated in Fig. 1b, the machine’s long-term functional performance is characterized by its steady-state statistics, governed by three types of observables [13]: the node probabilities $\pi_{i}$, the symmetric edge traffics [12] $\tau_{ij}=\pi_{i}k_{ij}+\pi_{j}k_{ji}$ where $k_{ij}$ is the transition rate from state i to j, and the antisymmetric edge net fluxes $J_{ij}=\pi_{i}k_{ij}-\pi_{j}k_{ji}$ spanned by the set of fundamental cycle fluxes $J_{c}$ (Kirchhoff’s current law) [23,24]. The goal is to control and optimize these observables by exploring the parameter space of transition rates $\left(k_{ij}\right)$.

In some situations, molecular machines are driven by differences across two or more equilibrium reservoirs and the flows are constrained by the Local Detailed Balance (LDB) condition [25–27]. This fixes the cycle affinity of any fundamental cycle c:

(1) $$\sum_{(ij)\in c}\text{ln}\frac{k_{ij}}{k_{ji}}=\frac{\Delta \mu -w}{k_{\text{B}}T},$$

where Δμ is the free energy consumption after completing the cycle and w is the work done by the cycle. To separate the controllable (tunable) degrees of freedom from these thermodynamic constraints, a common approach is to assume Arrhenius behavior of the transition rates [28]:

(2) $$k_{ij}=𝒩\text{exp}\left[-\left(B_{ij}-E_{i}\right)\right]\text{exp}\left(F_{ij}/2\right),$$

where 𝒩 is a global timescale prefactor, $E_{i}$ are node energies ($k_{\text{B}}T$ unit), $B_{ij}$ are kinetic edge barriers, and $F_{ij}$ are local edge affinities distributing the fixed cycle affinity. While this parameterization (Fig. 1c) has been useful in discovering various response relations [28–30], these parameters E,B, and F lack a conjugate structure with respect to the dynamical observables $\bar{x}=(\pi ,\tau ,J)$ (see SI Sec. I.A [31]). To get general design principles, resembling those that underlie the power of EQ thermodynamics, we must first identify the true conjugate forces of NEQ dynamics.

CFT derives the conjugate forces in terms of the maximization of the path entropy (Maximum Caliber) of the Markov dynamics [13, 32, 33]. The forces conjugate to the node distribution $\pi_{n}$, the edge traffic $\tau_{ij}$, and the cycle fluxes $J_{c}$ represent the affinities for node dwelling, edge exchange, and cycle completion, respectively (Fig. 1d):

(3) $$F_{\text{node},n}=\sum_{i(\neq m)}\left(k_{mi}-1\right)-\sum_{j(\neq n)}\left(k_{nj}-1\right),\;F_{\text{edge},ij}=\frac{1}{2}\text{ln}k_{ij}k_{ji},\;F_{\text{cycle},c}=\frac{1}{2}\sum_{(ij)\in c}\text{ln}\frac{k_{ij}}{k_{ji}}.$$

CFT handles the LDB constraint naturally as $F_{\text{cycle},c}$ is half of the cycle affinity, and these forces enable machine design.

### Conjugate coordinates solve constrained optimization.

Machine design first requires determining the optimal performance under physical constraints (e.g., maximizing speed under fixed free energy driving). CFT solves this using its conjugate coordinate systems and Legendre transformations [13]. By shifting from the observable coordinate (π,τ,J) to the observable-force coordinate $\left(\pi ,\tau ,F_{\text{cycle}}\right)$ (see SI Sec. I.A [31]), we can simply hold the environmental cycle forces $F_{\text{cycle}}$ constant while tuning the remaining parameters to meet functional targets and solve the underlying optimal rates $k_{ij}$. This has yielded the *Equal Traffic Principle* [13]—the rule that maximizing cycle flux in a unicircular process under a fixed total traffic budget requires maintaining equal traffic across all steps. Here, we apply this principle to diagnose the efficiency of the F_1_ molecular motor, extend it to design stronger enzyme inhibition, and show how to tune the speed, accuracy, and cost of kinetic proofreading networks.

### Caliber forces yield flow response rules.

The second task in machine design is to predict how tuning physical parameters affects the routing of network flows. CFT establishes far-from-EQ generalizations to Maxwell-Onsager symmetries and fluctuation-response equalities, mapping how dynamic observables respond to force perturbations [13, 14]. While these universal force principles do not require an Arrhenius assumption, biomolecular machines are physically tuned by evolution through their specific energy landscapes. By projecting these specific evolutionary knobs $\left(E_{n},B_{ij}\right)$ onto the general CFT forces (detailed in [14] and SI Sec. I.B [31]), we obtain two explicit design rules for flow routing via Arrhenius parameters.

1. **Node Energies affect the scaling of fluxes**: CFT shows that node energy perturbations isotropically scale the average value of any flux variable $\overset{‾}{\varphi }\in \left(\tau_{ij},J_{c}\right)$ (or their linear combinations):
(4) $$\frac{\partial \overset{‾}{\varphi }}{\partial E_{n}}=\pi_{n}\overset{‾}{\varphi }.$$
  The state (node) with highest $\pi_{n}$ is the most effective scaler, but pure $E_{n}$ perturbations cannot alter flux ratios. Thus, node energies alone cannot change the accuracy of kinetic proofreading networks [34]. This also yields a new scaling relation governing how node energy modulates the entropy production rate (EPR) of the network: $\partial_{E_{n}}\text{EPR}\equiv \partial_{E_{n}}\left[\sum_{c}J_{c}\left(2F_{\text{cycle},c}\right)\right]=\pi_{n}$ EPR where we have used $2F_{\text{cycle},c}=\sum_{(ij)\in c}\text{ln}\left(k_{ij}/k_{ji}\right)=\sum_{(ij)\in c}\text{arctanh}\left(J_{ij}/\tau_{ij}\right)$, confirming that its derivative with respect to $E_{n}$ vanishes.
2. **Kinetic Barriers affect the routing of fluxes**: Conversely, barrier perturbations $B_{ij}$ route fluxes, mediated by the edge’s net flux $J_{ij}$. For any distribution or flux observable ${\overset{‾}{x}}_{\alpha }\in \left(\pi_{n},\tau_{ab},J_{c}\right)$ (except the local traffic $\tau_{ij}$, which has an additional term in the equation [31]):
(5) $$\frac{\partial {\overset{‾}{x}}_{\alpha }}{\partial B_{ij}}=-J_{ij}A_{\alpha ,(ij)}^{-1}$$
  where $A^{-1}$ is the inverse of the force-rate Jacobian matrix $A_{(ij),\alpha }\equiv k_{ij}\partial_{k_{ij}}F_{\alpha }$, which can be easily evaluated based on Eqs. (3) [14, 31]. If an edge has zero net flux $\left(J_{ij}=0\right)$—such as those leading to deadend pathways—tuning its barrier alters only its own traffic $\tau_{ij}$ while leaving all other network observables invariant. We utilize this topological routing rule to establish the design limits of enzyme inhibitors.

These relationships constitute a comprehensive framework for molecular-machine *inverse design* (solving constrained optimal rates via conjugate coordinates) and forward *design* (routing flows via Arrhenius response rules). Here are three examples.

## 1) SPEEDING UP MOTORS THROUGH TRAFFIC CONTROL

The ${\text{F}}_{o}-{\text{F}}_{1}$ ATP synthase is biology’s rotary transducer: the ${\text{F}}_{o}$ motor converts the downhill flow of protons into mechanical torque, which drives the F_1_ motor to perform the energetically uphill synthesis of ATP. While earlier models evaluate its *thermodynamic efficiency* (work output versus free energy consumed) [35, 36], we show here that its speed and utility are also constrained by its *processive efficiency*, defined as the ratio of net transport to total transitions taken (J/τ). Here, we show that the F_1_ motor is limited by futile backstepping, a traffic imbalance that can only be corrected by routing flows via kinetic barriers.

**FIG. 2.**
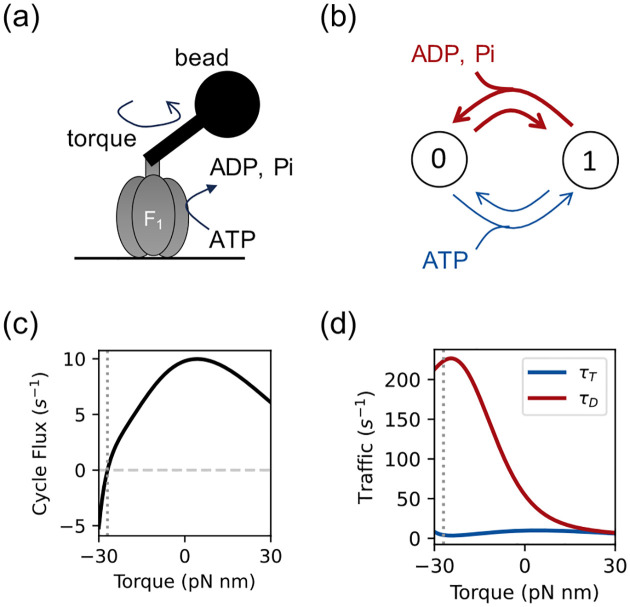
The F_1_-ATPase rotary motor properties. (a) In the Gerritsma and Gaspard assay, the motor is fixed to the surface and the bead rotates [15]. (b) The two-state Markov network: ATP binding (blue) and catalysis (red) transitions. The F_1_ motor rotates 120° every time a 0–1-0 (de-) hydrolysis cycle is completed. (c) The steady-state cycle flux, and (d) the symmetric traffics on the two edges versus the applied external torque. The traffic imbalance $\left(\tau_{D}\gg \tau_{T}\right)$ indicates the motor’s processive inefficiency *in vitro*.

To model the *in vitro* F_1_ motor assay (Fig. 2a), we adopt the 2-state, 2-edge network from Gerritsma and Gaspard [15] (Fig. 2b). Under the low-ATP conditions in the experiments (≈0.5μM), the slow ATP-binding rate justifies lumping the subsequent transitions. The motor transitions between an empty (node 0) and a nucleotide-occupied state (node 1) via the T transition (ATP binding/unbinding, ≈ 90° rotation) and the D transition (catalysis and product release, ≈ 30° rotation). The model fits well to the experimental data.

The network dynamics are governed by four loaddependent transition rates, $k_{\text{T}\pm }(\Gamma )$ and $k_{\text{D}\pm }(\Gamma )$, where Γ is the applied torque (see SI Sec. II.A [31]). We confine our analysis to Γ∈[-30,30] pN nm to preclude mechanical slipping and maintain the tight-coupling condition. These phenomenological rates satisfy the LDB cycle force constraint:

(6) $$\text{ln}\frac{k_{\text{T}+}(\Gamma )k_{\text{D}+}(\Gamma )}{k_{\text{T}-}(\Gamma )k_{\text{D}-}(\Gamma )}=2F_{\text{cycle}}=\frac{\Delta \mu +\Gamma \Delta \theta }{k_{\text{B}}T},$$

where Δθ=2π/3 is the rotation angle, and Δμ is the chemical free energy of ATP hydrolysis. Of interest are the steady-state distribution of the two states $\left(\pi_{0},\pi_{1}\right)$, the edge traffics ($\tau_{\text{T}},\tau_{\text{D}}$), and the cycle flux $\left(J_{c}\right)$ representing 1/3 of the motor’s physical rotation speed.

CFT’s observable-force coordinate $\left(\pi_{1},\tau_{\text{T}},\tau_{\text{D}},F_{\text{cycle}}\right)$ diagnoses the motor’s processive inefficiencies by decoupling the constrained free energy drive $\left(F_{\text{cycle}}\right)$ from the tunable kinetic variables. Under a fixed total kinetic activity budget $\left(\tau_{\text{tot}}=\tau_{T}+\tau_{D}\right)$, maximizing the cycle flux $J_{c}$ requires the traffic to be balanced across all steps $\left(\tau_{\text{T}}=\tau_{\text{D}}\right)$ [13]. This *Equal Traffic Principle* (ETP) bounds the motor’s processive efficiency $\left(J_{c}/\tau_{\text{tot}}\right)$ (see SI Sec. II.B [31]):

(7) $$\frac{J_{c}}{\tau_{\text{tot}}}\leq \frac{1}{2}\text{tanh}\left(\frac{F_{\text{cycle}}}{2}\right).$$

As shown in Fig. 2d, the *in vitro* rotor operates far below this optimum. The traffic imbalance $\left(\tau_{\text{D}}\gg \tau_{\text{T}}\right)$ indicates that kinetic activity is wasted on futile backstepping along the D transition.

To correct this imbalance, we compute the energetic response relations (Eqs. 4–5) with the analytical Jacobian inverse (see SI Sec. II.C [31]):

(8a) $$\frac{\partial \;\text{ln}\;J_{c}}{\partial E_{n}}=\frac{\partial \;\text{ln}\;\tau_{\text{T}}}{\partial E_{n}}=\frac{\partial \;\text{ln}\;\tau_{\text{D}}}{\partial E_{n}}=\pi_{n};$$

(8b) $$\frac{\partial \;\text{ln}\;J_{c}}{\partial B_{\text{T}}}=-\frac{\Sigma_{\text{D}}}{\Sigma },\frac{\partial \;\text{ln}\;J_{c}}{\partial B_{\text{D}}}=-\frac{\Sigma_{\text{T}}}{\Sigma },$$

where $\Sigma_{\text{X}}=k_{\text{X}+}+k_{\text{X}-}$ and Σ is the sum of all rates. These relations dictate that improving efficiency requires using the kinetic barriers ($B_{\text{T}},B_{\text{D}}$) as topological routers to balance the traffic. Conversely, perturbing the node energy $E_{n}$ only scales the fluxes by $\pi_{n}$, shifting state occupancies without altering the traffic ratio $\left(\tau_{\text{T}}/\tau_{\text{D}}\right)$ or the processive efficiency—a structural degeneracy inherently mapped by the $\left(\pi_{1},\tau_{T},\tau_{D},F_{\text{cycle}}\right)$ coordinate (Appendix II.B of SI [31]). Notably, these barrier sensitivities sum to −1, manifesting a cycle response symmetry [14] analogous to the Flux Control Summation Theorem in Metabolic Control Analysis [37, 38].

In summary, the *in vitro* F_1_-ATPase operates far from its optimal processive efficiency, due to considerable backstepping. While node energies scale the motor activity within a degenerate space of state occupancies, the kinetic barriers affect the routing and can correct this traffic imbalance.

## 2) DECOUPLING SPEED, ACCURACY, AND COST IN KINETIC PROOFREADING

Hopfield [17] and Ninio [16] were the first to explain how biomolecular processes can harness non-equilibrium driving to achieve certain biochemical process accuracies beyond the limits of thermodynamic equilibrium. For example, in *kinetic proofreading* (KP), a DNA polymerase enzyme copies DNA sequences onto new DNA with extremely small error rates, of one in 10^9^ monomers. Traditionally, KP is viewed as a trade-off among speed, accuracy, and energy cost [39, 40]. However, our design framework reveals that these canonical trade-offs are not absolute; they only manifest at the boundaries of the system’s kinetic capacity. Within the operational interior, these performance metrics can be effectively decoupled. We show here how kinetic barriers act as topological routers to circumvent these compromises, and remarkably, how real proofreading networks have evolved quite close to this optimal saturation point.

**FIG. 3.**
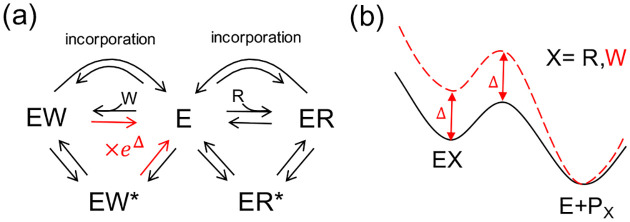
The classical model of kinetic proofreading assumes barrier asymmetry. (a) Traditional models assume that discriminating Right (R) from Wrong (W) relies solely on accelerating the unbinding of the W substrate, while keeping forward incorporation rates identical. (b) Because the Wrong bound state (red dashed) is destabilized by a node energy Δ, maintaining identical incorporation rates mathematically requires elevating the Wrong kinetic barrier by exactly the same Δ.

The topological necessity of barrier routing is embedded in foundational KP models [16, 17]. Classic models attributed error correction primarily to differing unbinding rates for the Right (cognate) and Wrong (non-cognate) substrates, while assuming identical forward incorporation rates (Fig. 3a). However, applying CFT response rules reveals a hidden topological constraint. As established earlier, node energies $\left(E_{n}\right)$ only scale fluxes ($\partial \;\text{ln}\;J_{c}/\partial E_{n}=\pi_{n}$). Thus, node energy differences alone cannot alter the error rate $\left(\varepsilon ={\text{log}}_{10}\left(J_{\text{W}}/J_{\text{R}}\right)\right)$, as independently reported by others [34]. To maintain identical incorporation rates while altering unbinding rates, the classic model implicitly requires asymmetric incorporation barriers (Fig. 3b). Barrier routing, not just binding affinity, is the engine of proofreading.

To move beyond idealized symmetric models, we apply our framework to the asymmetric networks of real KP systems mapped by recent single-molecule experiments: the E. coli ribosome and T7 DNA polymerase [18, 19].

### Performance trade-offs emerge only at the parameterspace boundaries.

**FIG. 4.**
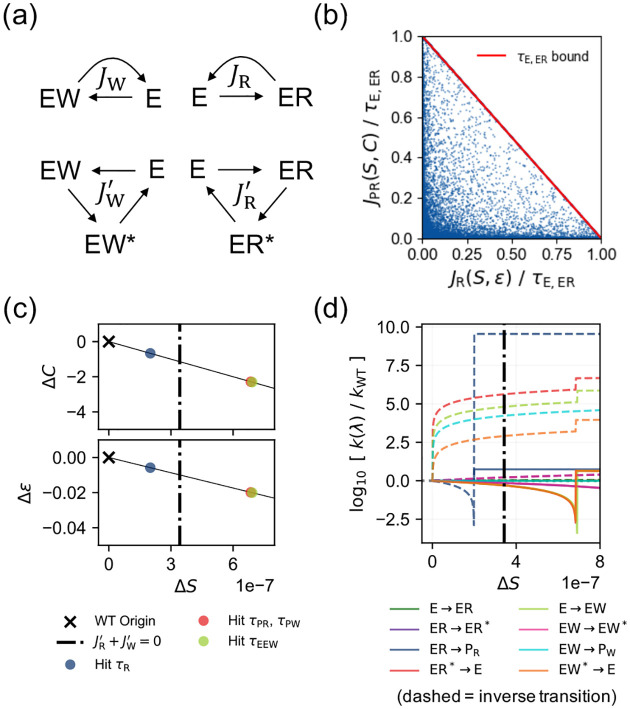
Inverse design of the T7 DNA polymerase. (a) The four fundamental cycles defining the incorporation $\left(J_{\text{R}},J_{\text{W}}\right)$ and proofreading $\left(J_{\text{R}}^{\prime },J_{\text{W}}^{\prime }\right)$ fluxes. (b) Validating the trade-off bound in Eq. (9) where $J_{\text{R}}$ and $J_{\text{R}}^{\prime }$ are functions of the speed (S), accuracy (ε), cost (C). See main text for expressions. Dots are generated via 10^5^ log-uniform traffic (τ) values $\in \left(10^{-3},10^{5}\right)$. Bound saturates when $J_{\text{R}}+J_{\text{R}}^{\prime }=\tau_{\text{E},\text{ER}}\Leftrightarrow p_{\text{ER},\text{E}}=0$. (**c**) An inversely-designed “evolutionary” process increasing speed S while reducing error ε and cost C from the wild-type origin (black cross). $\Delta Z=Z-Z_{\text{WT}}$ represents the excess to wild-type values (Z=C,ε,S). Colored dots indicate where specific traffic limits $\left(\sum J\leq \tau \right)$ saturate and are enlarged tenfold to allow further optimization. The vertical dash-dotted line marks the transition into the anti-proofreading regime ($J_{\text{W}}^{\prime }+J_{\text{R}}^{\prime }\leq 0$). (d) Solving the driving protocol of transition rates realizing the linear inverse design path in (c). It requires speeding up various reversed rates (colored dashed line). Jumps reflects the traffic limits relaxation algorithm.

We use CFT’s coordinate systems to show that speed-accuracy-cost trade-offs are boundary constraints. To demonstrate this in the T7 DNA polymerase, we formalize the network’s performance using four fundamental cycle fluxes (Fig. 4a): correct and incorrect incorporations $\left(J_{R},J_{W}\right)$ and their corresponding proofreading discards ($J_{\text{R}}^{\prime },J_{\text{W}}^{\prime }$). The performance metrics are algebraic combinations of these fluxes: total incorporation speed $S=J_{\text{R}}+J_{\text{W}}$, error $\varepsilon ={\text{log}}_{10}\left(J_{\text{W}}/J_{\text{R}}\right)$, and power cost $C=S\Delta \mu_{\text{p}}+\left(J_{\text{R}}^{\prime }+J_{\text{W}}^{\prime }\right)\Delta \mu_{\text{NTP}}$, where $\Delta \mu_{\text{p}}$ and $\Delta \mu_{\text{NTP}}$ are the free energies of phosphodiester bond formation and NTP hydrolysis.

We use CFT’s canonical coordinate $z=\left(\pi ,\tau ,F_{\text{cycle}}\right)$ to map these performance targets back to the underlying transition rates. Since each fundamental cycle is identified by a defining edge, or “chord”, we swap the traffic variables on these chords for the fundamental cycle fluxes $J_{\text{cycle}}$, leaving the traffics on all other edges as the remaining vector $\tau_{\text{rem}}$. Expressing these cycle fluxes in terms of our target metrics establishes the functional coordinate $\left(\pi ,\tau_{\text{rem}},S,\varepsilon ,C,r^{\prime },F_{\text{cycle}}\right)$, where $r^{\prime }=J_{\text{W}}^{\prime }/J_{\text{R}}^{\prime }$ is the discard ratio. This yields an exact analytical map $k_{ij}\left(\pi ,\tau_{\text{rem}},S,\varepsilon ,C,r^{\prime },F_{\text{cycle}}\right)$ that translates target performances and cycle force constraints directly into the required transition rate configurations (see SI Sec. III.A [31]).

This parameterization yields physical performance bounds dictated by the non-negativity of one-way transition fluxes $\left(p_{ij}=\left[\tau_{ij}+J_{ij}\right]/2\geq 0\right)$. For example, the kinetic capacity of the initial binding step of the Right substrate imposes the constraint $J_{\text{R}}(S,\varepsilon )+J_{\text{R}}^{\prime }\left(S,C,r^{\prime }\right)\leq \tau_{\text{E},\text{ER}}$ (i.e., $p_{\text{ER}\to \text{E}}\geq 0$). This yields the inequality:

(9) $$\frac{S}{1+10^{\varepsilon }}+\frac{C-S\Delta \mu_{p}}{\Delta \mu_{\text{NTP}}\left(1+r^{\prime }\right)}\leq \tau_{\text{E},\text{ER}}.$$

This bound (numerically verified in Fig. 4b) describes a traffic jam effect: productive incorporation (the first term) and futile proofreading (the second term) must compete for a finite kinetic bandwidth $\left(\tau_{\text{E},\text{ER}}\right)$. Because S,ε, and C serve as independent coordinate dimensions, a trade-off manifests *only* at the boundary where this inequality saturates. As long as the traffic bandwidth is not saturated (the feasible interior), these metrics are decoupled. An enzyme can simultaneously improve speed, reduce error, and lower cost under fixed thermodynamic cycle drives $\left(F_{\text{cycle}}\right)$. Fig. 4c demonstrates this by designing a linear trajectory that optimizes all three metrics from their wild-type origins. Whenever this designed path saturates a local traffic bound ($p_{ij}\to 0$, marked with dots), we iteratively relax that specific $\tau_{ij}$ limit tenfold, allowing the optimization to continue (see SI Sec. III.C for algorithmic details [31]).

At some point, simultaneously increasing speed and decreasing cost forces the proofreading fluxes to become negative $\left(J_{\text{R}}^{\prime }+J_{\text{W}}^{\prime }\leq 0\right)$. To sustain a lower cost, the system must synthesize NTP, entering an “anti-proofreading” regime (crossing the vertical dash-dotted line in Fig. 4c, d) [41]. By feeding this targeted linear performance path back into our inverse map $k_{ij}\left(\pi ,\tau_{\text{rem}},S,\varepsilon ,C,r^{\prime },F_{\text{cycle}}\right)$, we calculate the driving protocol of transition rates required to realize it (Fig. 4d). Achieving this regime necessitates accelerating reversed transition rates (colored dashed lines in Fig. 4d) to drive the proofreading cycle backwards. Because the parameters π and $r^{\prime }$ remain freely tunable, the shown driving protocol is not unique—demonstrating a flexibility CFT uncovers for the design of kinetic proofreaders.

### Barrier routing reveals an evolutionary “free lunch”.

While the inversely designed protocol proves that decoupling speed, error, and cost is mathematically possible, applying CFT’s response rules reveals a simpler physical implementation: this optimal routing can be achieved by tuning only specific kinetic barriers. Furthermore, evaluating the wild-type parameters demonstrates that biological evolution has already driven this specific tuning mechanism to near-saturation.

**FIG. 5.**
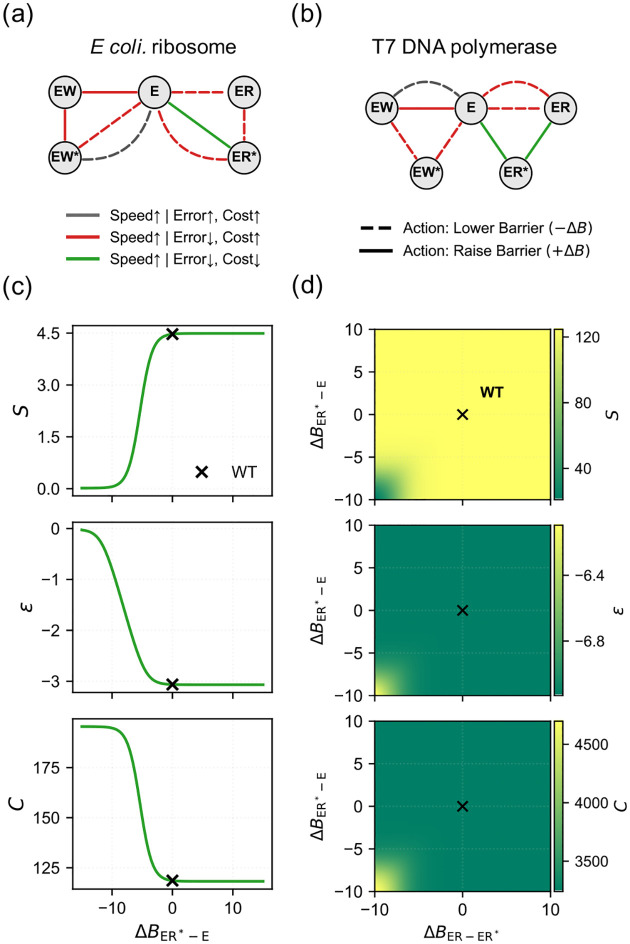
Performance routing and evolutionary saturation in kinetic proofreading. **(a, b)** Barrier sensitivity maps for T7 DNA polymerase (a) and *E. coli* ribosome (b). Colors indicate the different types of responses for each barrier. Solid and dashed lines denote raising (+ΔB) and lowering (-ΔB) barriers to raise speed, respectively. (c, d) Performance metrics (S,ε,C) along the “free lunch” barrier tuning (Green in a,b). The wild-type (WT) parameters (marked as x) reside near the saturating plateau for all three metrics.

Using our analytical barrier routing rules (Eq. 5), we map the performance gradients for T7 DNA polymerase and the *E. coli* ribosome (Fig. 5a, b). This reveals three tuning directions. Most barrier perturbations enforce a standard trade-off (red): speed increases at the expense of higher energetic cost. Accelerating the Wrong incorporation step (gray) is detrimental, compounding both error and cost. Crucially, both networks share a topological “free lunch” direction (green) that simultaneously increases speed while lowering error and cost.

In both systems, this optimal protocol is accessed by raising the barrier in the discard pathway of the Right substrate. This theoretical requirement provides a physical rationale for the asymmetric rates observed in real proofreading networks [18, 19]—suggesting that evolution has developed structural mechanisms to selectively tune the cognate discard barrier without symmetrically affecting the non-cognate one.

Tracking the performance metrics along this specific path—the ${\text{ER}}^{*}⇌\text{E}$ transition in the ribosome, and the corresponding excision pathway in the polymerase—allows us to directly interrogate the wild-type enzymes. As illustrated in Fig. 5c and d, the wild-type parameters reside near the plateau where the marginal gain of further raising this barrier almost vanishes. Biological evolution appears to have pushed this specific topological router to its optimal limit.

In summary, the canonical understanding of speed-accuracy-cost trade-offs in kinetic proofreading is boundary constraints [39, 40, 42, 43]. Within the operational interior, these performance metrics can be decoupled and inversely designed. This is consistent with the emerging realization that NEQ driving can circumvent static thermodynamic trade-offs [44]. Evolution appears to have navigated this interior region by pushing the cognate discard barrier along a “free lunch” tuning direction toward near-saturation.

## 3) STRONGER ENZYME INHIBITION: DEAD-ENDS VERSUS LEAKY LOOPS

Classical pharmacology evaluates enzyme inhibitor efficacy based on binding affinities, $\text{ln}\left(k_{\text{on}}/k_{\text{off}}\right)$ of the inhibitor molecule to the target enzyme [21]. By treating Michaelis-Menten (MM) inhibition as a NEQ flow-routing problem, we demonstrate that inhibitor efficacy fundamentally depends on network topology. To analyze these networks, we must first address the non-invertible catalytic step (ES → E + P). We extend CFT—originally formulated for networks where all transitions possess non-zero inverse rates—directly to this zero-rate limit. Because the catalytic transition is non-invertible, its symmetric traffic equals its directed flux $\left(\tau_{\text{cat}}=J_{\text{cat}}\right)$. By selecting a spanning tree where this non-invertible edge acts as a fundamental cycle chord, maximizing the path entropy (Maximum Caliber) reveals that most conjugate forces preserve their original forms (see SI Sec. IV.A [31]). The single modification occurs on the catalytic cycle: the lack of an inverse transition alters its localized force contribution from $\frac{1}{2}\text{ln}\left(k_{+}/k_{-}\right)$ to $\text{ln}k_{\text{cat}}$, yielding the cycle force:

(10) $$F_{\text{cat}}=\text{ln}\;k_{\text{cat}}+\frac{1}{2}\text{ln}\;\left(\frac{k_{\text{E},\text{ES}}}{k_{\text{ES},\text{E}}}\right).$$

With this observable-force conjugacy established, CFT’s analytical machinery remains valid for MM networks. We can now use it to evaluate the classical inhibition archetypes.

**FIG. 6.**
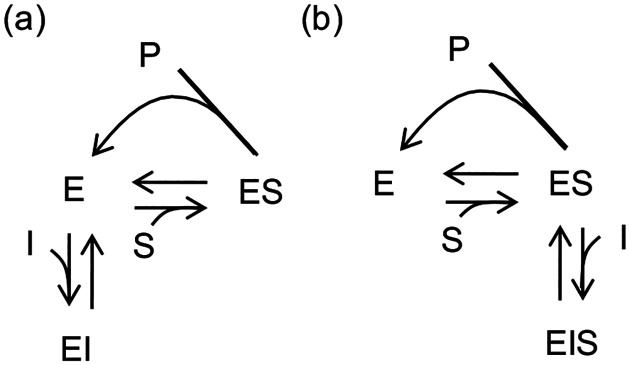
Competitive and uncompetitive inhibitors rely on topological dead-ends. Network diagrams for (a) competitive and (b) uncompetitive inhibition. Unlike the noncompetitive loop, the inhibitor-bound states (EI and EIS) form dead-end pathways carrying zero steady-state net flux. This topological distinction renders their long-term catalytic rates insensitive to kinetic barrier perturbations along the inhibitor pathways.

We first demonstrate that competitive and uncompetitive inhibitors act as topological dead-ends optimized solely by node energies (Fig. 6). The inhibitor-bound states (EI and EIS) form dead-end pathways that carry zero net flux $\left(J_{\text{sideway}}=0\right)$. According to CFT’s barrier routing rule, tuning the kinetic barriers along these pathways has zero effect on the long-term catalytic production. Therefore, to optimize dead-end inhibitors, designers cannot rely on barrier routing; they must instead deepen the node energy ($E_{n}$) of the bound state, maximizing the binding affinity purely by suppressing $k_{\text{off}}$.

**FIG. 7.**
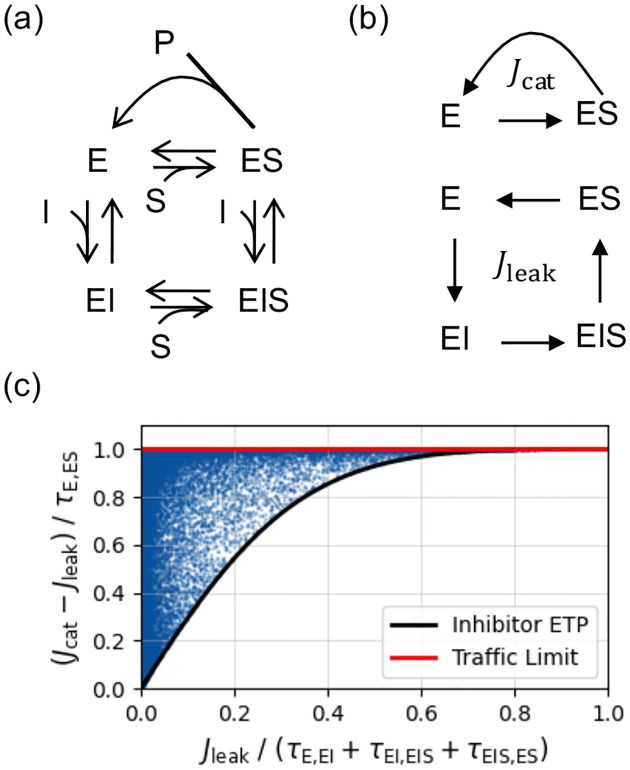
Inhibitor Equal Traffic Principle in noncompetitive enzyme inhibition. (a) The four-state network with a non-invertible catalytic step (ES→E+P). (b) The two fundamental cycle fluxes: the non-invertible catalytic flux $\left(J_{\text{cat}}\right)$ and the invertible leak flux $\left(J_{\text{leak}}\right)$. (c) Numerical validation of the operational bounds. The blue scatter points represent 10^5^ randomly sampled kinetic networks with log-uniform transition rates spanning six orders of magnitude, constrained to maintain a futile inhibitor loop $\left(F_{\text{leak}}=0\right)$. For a given $J_{\text{leak}}$, catalytic production is minimized when kinetic traffic is equally distributed across the sideway edges of the inhibitor loop (lower bound). Production is maximized when the reverse one-way flux to the free enzyme vanishes ($p_{\text{ES},\text{E}}\to 0$, upper bound).

In contrast, noncompetitive inhibitors form leaky loops that require kinetic barrier routing for optimization. These inhibitors form a closed loop (E⇌EI⇌EIS⇌ES⇌E) that couples to the catalytic step (Fig. 7a). Although this internal futile cycle has zero cycle force $\left(F_{\text{leak}}=0\right)$, the non-invertible catalysis continually depletes ES and replenishes E. This tilted population gradient acts as a pump, driving a compensatory clockwise leak flux $\left(J_{\text{leak}}>0\right)$ through the indirect inhibitor pathway (see SI Sec. IV.B for proof [31]). Because of this non-zero leak flux, the kinetic barriers act as effective control knobs for noncompetitive inhibition.

To optimize this network, we derive the Inhibitor Equal *Traffic Principle* (iETP) as a multicircular generalization to ETP. Using the CFT coordinate $z=\left(\pi ,\tau ,J_{\text{leak}},F_{\text{leak}}\right)$, we express the catalytic production [31]:

(11) $$J_{\text{cat}}=J_{\text{leak}}+\tau_{\text{E},\text{ES}}\;\text{tanh}\left({𝒜}_{\text{side}}\right),$$

where ${𝒜}_{\text{side}}=\sum_{\eta }\text{arctanh}\left(J_{\text{leak}}/\tau_{\eta }\right)$ is the total affinity across the three sideway edges of the loop. Minimizing $J_{\text{cat}}$ under fixed traffic τ requires minimizing ${𝒜}_{\text{side}}$, which dictates equalizing the traffic across these sideway edges. Therefore, maximizing inhibition requires a balanced traffic distribution across the leak loop. Conversely, the maximum catalytic production occurs at the traffic upper bound $\left(J_{\text{cat}}^{\text{max}}=J_{\text{leak}}+\tau_{\text{E,ES}}\right)$, where the reverse one-way flux $p_{\text{ES,E}}$ vanishes. We numerically validate these bounds in Fig. 7c.

In summary, extending CFT to catalytic networks recasts enzyme inhibition from an EQ affinity problem into a NEQ topological flow-routing problem. Competitive and uncompetitive inhibitors act as topological dead-ends, blind to barrier routing and reliant entirely on node energy optimization. In contrast, noncompetitive inhibitors form leaky loops, reaching maximum efficacy only when kinetic barriers actively balance their internal traffic. This establishes a NEQ physical basis for targeted drug design.

## DISCUSSION

This work reframes the design of nonequilibrium molecular machines as a flow-routing problem. Traditional thermodynamic models focus on energetic drives, net fluxes, and correlating machine performance with entropy production, but they lack explicit handles for the *topological misflows* that degrade machine efficiency—such as futile backstepping in the F_1_ motor, branched misrouting in kinetic proofreading, or dead-ends or leaks in enzyme inhibition. By pairing dynamic observables with conjugate path-entropic forces, Caliber Force Theory (CFT) establishes a dual design framework: *inverse design* to map performance targets to optimal transition rate configurations, and *forward design* to scale or route flows with node energies or kinetic barriers.

While we focused on biochemical networks governed by Local Detailed Balance and Arrhenius energetics, the framework is independent of these specific physical assumptions. Inherited from its path-entropy-based construction [13], CFT Design applies to any ergodic Markov jump process and can be generalized to continuous processes in principle. This extends the applicability to other nonequilibrium systems, such as ecological population dynamics [45] or vehicular traffic [13]. Just as equilibrium thermodynamics provides the foundational conjugate forces for static systems, CFT Design provides them for optimizing and controlling nonequilibrium flows.

## Supplementary Material

1

## Data Availability

The codes used to produce the Figures are available at DOI: 10.5281/zenodo.20057619.
