# Supplementary material for: Nonequilibrium Theory for Molecular Machine Design
Source: ArXiv. 2026 May 9:arXiv:2605.09105v1. Preprint. [Version 1] (PMC13178445)
Supplement: 1 [file NIHPP2605.09105V1-supplement-1.pdf]

Supplementary Information for: "Nonequilibrium Theory for Molecular Machine Design"

Ying-Jen Yang[1,*] and Ken A. Dill[1,2,3]

[1] *Laufer Center of Physical and Quantitative Biology, Stony Brook University*
[2] *Department of Physics and Astronomy, Stony Brook University*
[3] *Department of Chemistry, Stony Brook University*

[*] ying-jen.yang@stonybrook.edu

(Dated: May 12, 2026)

CONTENTS

I. A Brief Review of Caliber Force Theory (CFT) 1
A. Conjugate Coordinates and the Exponential Family 1
B. General Response Relations and Symmetries 4
1. Node Energy as an Isotropic Scaler 5
2. Kinetic Barrier as a Topological Router 6

II. Example 1: F_1-ATPase Molecular Rotors 6
A. The Gerritsma and Gaspard (2010) model and implementation details 6
B. Equal Traffic processivity bound and inverse design 7
C. Response Relations and Schur Complement Inversion 9

III. Example 2: Kinetic Proofreading and the Performance Envelope 10
A. Construction of mixed coordinates and rate mapping 10
B. Traffic bounds and anti-proofreading regimes 12
C. Algorithmic details of the linear exploration 13

IV. Example 3: Enzyme Inhibition and Non-Invertible Networks 13
A. Caliber Force construction for non-invertible networks 13
B. Explicit derivation of the Inhibitor Equal Traffic Principle 15

I. A BRIEF REVIEW OF CALIBER FORCE THEORY (CFT)

The operational power of equilibrium (EQ) thermodynamics stems from its conjugate structure of extensive observables and intensive forces, formally linked by Legendre transforms. The origin of the conjugacy lies in the Boltzmann-Gibbs distribution as an exponential family maximizing state entropy (Max Ent). The resulting zoo of conjugate coordinates allows the handling of macroscopic constraints (e.g., isothermal, isobaric, isochoric) via ensemble transformations to the most suitable coordinate system (e.g., canonical, microcanonical). This mathematical structure exists because variables like $1/T$, p/T, and $-\mu/T$ are defined as entropic forces (derivatives of the state entropy potential), ensuring they are conjugate to the physical observables U, V, and N. Caliber Force Theory (CFT) is designed to mirror this exact conjugate structure for nonequilibrium (NEQ) flows within a network state space. In this section, we recapitulate the foundations of CFT, detailing the construction of conjugate coordinates via Maximum Caliber [13], the derivation of the Response-Inverse-Matrix (RIM) relation [14], and the explicit proofs for the Arrhenius energetic perturbation rules utilized in the main text.

A. Conjugate Coordinates and the Exponential Family

Consider a continuous-time Markov jump process on a network state space consisting of N nodes and M edges, parameterized by transition rates k_{ij} (from i to j). We use the terminology that a transition is "invertible" if $k_{ij} > 0 \iff k_{ji} > 0$, and a system is "reversible" if it obeys detailed balance. We here assume all transitions are invertible (we will generalize this for non-invertible systems in Sec. IV), and the system can be either reversible or irreversible. The steady-state statistics of this system are fully described by the state probabilities π_i and the one-way probability fluxes $p_{ij} = \pi_i k_{ij}$. Because elementary transitions $i \mapsto j$ are governed by independent Poisson processes, specifying these average observables entirely fixes the

transition rates via $k_{ij} = p_{ij}/\pi_i$, thereby determining the full dynamics and all higher-order statistics.

To construct a "microcanonical-like" observable coordinate for dynamics, the chosen set of observables must be linearly independent. Raw transition counts and dwell times are degenerate due to probability conservation laws: probabilities must normalize ($\sum_i \pi_i = 1$), and steady-state fluxes must satisfy Kirchhoff's current law ($\sum_{j(\neq i)} p_{ij} = \sum_{j(\neq i)} p_{ji}$). CFT resolves this by projecting the dynamics onto a complete, non-degenerate observable basis.

Let N_{ij} denote the number of $i \to j$ transitions and T_n the dwell time in state n over a trajectory of duration t. The fundamental counting observables are defined as $\mathbf{X} = (T_n, \Phi_{ij}, \Psi_c)$, where $\Phi_{ij} = N_{ij} + N_{ji}$ is the symmetric edge traffic count, and $\Psi_c = N_{ab} - N_{ba}$ is the antisymmetric net flux count across the defining chord ab of fundamental cycle c. Their corresponding empirical rates are $\mathbf{x} = \mathbf{X}/t = (f_n, \phi_{ij}, \psi_c)$, whose long-term steady-state averages yield the observable basis $\bar{\mathbf{x}} = (\pi_n, \tau_{ij}, J_c)$:

1. **Node probabilities (π_n):** The state occupancies, yielding $N-1$ independent variables by excluding an arbitrary reference node m to account for normalization.

2. **Edge traffics (τ_{ij}):** The symmetric, total kinetic activity on each edge, defined as $\tau_{ij} = p_{ij} + p_{ji}$, yielding M variables (a term first coined by C. Maes and collaborators [12]).

3. **Fundamental cycle fluxes (J_c):** The antisymmetric net fluxes $J_{ij} = p_{ij} - p_{ji}$ are degenerate due to Kirchhoff's current law. A standard method to find a linearly independent basis is to select a spanning tree of the network [23, 24]. Each chord (edge ab) added to the tree defines a fundamental cycle c, and its net flux $J_c = J_{ab} = \pi_a k_{ab} - \pi_b k_{ba}$ serves as an independent basis vector spanning all steady-state net flows. In network topology, a spanning tree leaves exactly $M - N + 1$ chords.

Together, these form a complete, linearly independent "microcanonical-like" coordinate system of exactly $2M$ dynamical observables $\bar{\mathbf{x}} = (\boldsymbol{\pi}, \boldsymbol{\tau}, \boldsymbol{J})$—comprising $(N-1) + M + (M - N + 1) = 2M$ dimensions—which parameterize each rate as $k_{ij}(\bar{\mathbf{x}}) = [\tau_{ij} + J_{ij}(J_c)]/(2\pi_i)$.

The Dimensionality Mismatch of Arrhenius Energetics: To establish a formal framework for design, we seek a set of force variables strictly **conjugate** to these $2M$ observables, ensuring a one-to-one mapping and enabling coordinate swapability via Legendre transforms. One approach is to parameterize the rates using Arrhenius energetics: $k_{ij} = \mathcal{N} \exp[-(B_{ij} - E_i)] \exp(F_{ij}/2)$. However, for a connected network with N nodes and M edges, this parameterization utilizes N node energies (E_n), M kinetic barriers (B_{ij}), and M edge affinities (F_{ij}), totaling $N + 2M$ energetic parameters.

Because the true kinetic degrees of freedom defining the Markov jump process are strictly $2M$, this fundamental dimensionality mismatch implies there are exactly N degenerate dimensions in the Arrhenius parameterization. This gauge degeneracy can be demonstrated by introducing an arbitrary state potential ϕ_i. If we shift the node energies by this potential ($E_i \to E_i + \phi_i$), we can perfectly absorb this shift and keep all transition rates k_{ij} mathematically invariant by simultaneously adjusting the barriers and local affinities:

$$B_{ij} \to B_{ij} + \frac{\phi_i + \phi_j}{2}, \tag{S1}$$

$$F_{ij} \to F_{ij} + \phi_j - \phi_i. \tag{S2}$$

Because $\sum_{(ij)\in c}(\phi_j - \phi_i) = 0$ around any closed loop, the thermodynamic cycle affinities ($\sum_c F_{ij}$) remain invariant under this gauge transformation.

This mathematical degeneracy proves that infinite combinations of E, B, and F map to the exact same dynamics. Consequently, they cannot form a one-to-one conjugate coordinate system with the observables $\bar{\mathbf{x}}$. Logically, this is expected: Arrhenius parameters describe specific energetic properties of a physical barrier-crossing model, whereas the observables $(\boldsymbol{\pi}, \boldsymbol{\tau}, \boldsymbol{J})$ describe the purely dynamical properties of the generic Markov process. One should not expect model-specific properties to serve as the generic dynamical conjugates. The true conjugate forces must emerge from the dynamics itself.

To establish a Legendre-dual parameterization for these dynamical observables and obtain the force coordinate variables, CFT employs the Principle of Maximum Caliber (Max Cal) [13, 33], which is to NEQ dynamics what Max Ent is to EQ distributions. Let $\mathcal{P}_{\mathbf{k}}(\omega_t)$ be the probability of a path ω_t of duration t generated by rates $\mathbf{k}$. Max Cal maximizes the relative path entropy rate, $\mathfrak{s}_{\text{path}} = \lim_{t\to\infty} -\frac{1}{t}\sum_{\omega_t} \mathcal{P}_{\mathbf{k}}(\omega_t) \ln[\mathcal{P}_{\mathbf{k}}(\omega_t)/\mathcal{P}_{\mathbf{u}}(\omega_t)]$, relative to a uniform reference "doldrum" process ($u_{ij} = 1$), subject to constraints on the average observables $\bar{\mathbf{x}}$.

This constrained optimization generates a dynamic *exponential family* of path probabilities:

$$\mathcal{P}_{\mathbf{k}}(\omega_t) = \mathcal{P}_{\mathbf{u}}(\omega_t)\frac{\exp\left(\mathfrak{F}\cdot\mathbf{X}(\omega_t)\right)}{\mathcal{Z}_t(\mathfrak{F})}, \tag{S3}$$

where $\mathcal{Z}_t(\mathfrak{F})$ is the dynamic partition function. A generic additive path observable takes the form $\int_0^t \mathbf{f}(i_s)ds + \sum_l \mathbf{g}(i_{t_l^-}, i_{t_l^+})$, where s denotes continuous time and l indexes the discrete jumps. The basis $\mathbf{X}$ is constructed by choosing specific indicator functions for $\mathbf{f}$ and $\mathbf{g}$: node dwell times use the Kronecker delta indicator $\mathbf{f}(x) = \delta_{x,n}$ (with $\mathbf{g} = 0$); edge traffic counts use $\mathbf{g}(i,j) = \delta_{i,a}\delta_{j,b} + \delta_{i,b}\delta_{j,a}$ (with $\mathbf{f} = 0$); and cycle net flux counts use $\mathbf{g}(i,j) = \delta_{i,a}\delta_{j,b} - \delta_{i,b}\delta_{j,a}$ on the defining chord ab.

The Lagrange multipliers $\mathfrak{F}$ conjugate to these constraints are exactly the *path entropic forces*. In the long-term limit, the scaled cumulant generating function—acting as the NEQ free energy or log dynamic partition function, defined as the Caliber rate $\mathfrak{c}(\mathfrak{F}) = \lim_{t\to\infty}\frac{1}{t}\ln\mathcal{Z}_t(\mathfrak{F})$—is obtained via the Legendre-Fenchel transform [46]:

$$\mathfrak{c}(\mathfrak{F}) = \max_{\bar{\mathbf{x}}}\left[\mathfrak{s}_{\text{path}}(\bar{\mathbf{x}}) + \mathfrak{F}\cdot\bar{\mathbf{x}}\right]. \tag{S4}$$

This maximization explicitly defines the coordinate transformation from $\bar{\mathbf{x}}$ to $\mathfrak{F}$, guaranteeing that the forces are derivatives of the entropy: $\mathfrak{F} = -\partial\mathfrak{s}_{\text{path}}/\partial\bar{\mathbf{x}}$. Conversely, the inverse coordinate transform from $\mathfrak{F}$ back to $\bar{\mathbf{x}}$ is given by:

$$-\mathfrak{s}_{\text{path}}(\bar{\mathbf{x}}) = \max_{\mathfrak{F}}\left[\mathfrak{F}\cdot\bar{\mathbf{x}} - \mathfrak{c}(\mathfrak{F})\right]. \tag{S5}$$

This guarantees that the Caliber function is a generating function, $\bar{\mathbf{x}} = \partial\mathfrak{c}/\partial\mathfrak{F}$. The standard Legendre transform expression, $\mathfrak{c} = \mathfrak{s}_{\text{path}}(\bar{\mathbf{x}}) + \mathfrak{F}\cdot\bar{\mathbf{x}}$, serves as a compressed summary of these two explicit optimizations: when evaluated in the $\mathfrak{F}$ coordinate, it reflects Eq. (S4), and when evaluated in the $\bar{\mathbf{x}}$ coordinate, it reflects Eq. (S5).

A key advantage of this conjugate structure is the ability to construct other coordinates via partial Legendre transforms. For example, to parameterize the dynamics under a fixed cycle force $\mathfrak{F}_{\text{cycle}}$ while treating the internal kinetic layout (probabilities and traffics) as independent variables, one constructs the potential $\mathcal{F}(\pi_n, \tau_{ij}, \mathfrak{F}_{\text{cycle}})$:

$$\mathcal{F}(\pi_n, \tau_{ij}, \mathfrak{F}_{\text{cycle}}) = \max_{J_c}\left[\mathfrak{s}_{\text{path}}(\bar{\mathbf{x}}) + \mathfrak{F}_{\text{cycle}}\cdot J_c\right]. \tag{S6}$$

This directly mirrors the transformation from the microcanonical ensemble (U,V,N) to the canonical one (T,V,N) by the Helmholtz free energy $F(T,V,N)$ in equilibrium thermodynamics. The inverse partial Legendre-Fenchel transform then reveals how to compute the flux J_c directly from the observable-force coordinate:

$$-\mathfrak{s}_{\text{path}}(\bar{\mathbf{x}}) = \max_{\mathfrak{F}_{\text{cycle}}}\left[\mathfrak{F}_{\text{cycle}}\cdot J_c - \mathcal{F}(\pi_n, \tau_{ij}, \mathfrak{F}_{\text{cycle}})\right], \tag{S7}$$

which guarantees $J_c = \partial\mathcal{F}/\partial\mathfrak{F}_{\text{cycle}}$. Other mixed coordinates can be constructed similarly. We will explicitly deploy this mixed-coordinate strategy in the subsequent sections to derive the Equal Traffic Principle (ETP) for molecular motors, the operational envelopes of kinetic proofreading, and the inhibitor generalizations of the ETP for enzyme inhibition.

To give these path entropic forces more concrete physical meanings, CFT solves for their transition rate expressions. CFT utilizes Large Deviation Theory (LDT) [47] to evaluate the Caliber via the eigenvalue problem of a "tilted matrix" $\tilde{\mathbf{M}}(\mathfrak{F})$. The off-diagonal elements are tilted by the forces associated with the transitions, $\tilde{M}_{ij} = \exp(\mathfrak{F}\cdot\mathbf{g}(i,j))$, while the diagonal elements capture node constraints, $\tilde{M}_{ii} = -N_i + \mathfrak{F}\cdot\mathbf{f}(i)$, where N_i is the out-degree of node i (the number of connected neighbors) in the reference doldrum process. The largest eigenvalue of $\tilde{\mathbf{M}}$ is exactly the Caliber rate $\mathfrak{c}$, and the transition rates k_{ij} are determined by the forces and the corresponding right eigenvectors v_i of $\tilde{\mathbf{M}}$:

$$k_{ij}(\mathfrak{F}) = \frac{v_j(\mathfrak{F})}{v_i(\mathfrak{F})}e^{\mathfrak{F}\cdot\mathbf{g}(i,j)} \quad (i \neq j), \quad \text{and} \tag{S8a}$$

$$k_{ii}(\mathfrak{F}) = -N_i + \mathfrak{F}\cdot\mathbf{f}(i) - \mathfrak{c}(\mathfrak{F}). \tag{S8b}$$

These relations map the forces back to the transition rates.

By taking the logarithmic ratios of these transition rates along specific topological structures—such as the product of forward and backward rates on an edge, or the product of rates around a closed loop—the eigenvector ratios v_j/v_i perfectly telescope

and cancel out. This algebraic cancellation yields the explicit expressions for the conjugate forces $\mathfrak{F} = (\mathfrak{F}_{\text{node},n}, \mathfrak{F}_{\text{edge},ij}, \mathfrak{F}_{\text{cycle},c})$ directly in terms of the rates [13]:

$$\mathfrak{F}_{\text{node},n} = \sum_{i(\neq m)} (k_{mi} - 1) - \sum_{j(\neq n)} (k_{nj} - 1), \tag{S9}$$

$$\mathfrak{F}_{\text{edge},ij} = \frac{1}{2} \ln k_{ij} k_{ji}, \tag{S10}$$

$$\mathfrak{F}_{\text{cycle},c} = \frac{1}{2} \sum_{(ij)\in c} \ln \frac{k_{ij}}{k_{ji}}. \tag{S11}$$

Deriving these explicit rate expressions is a critical theoretical step. It grounds the path entropic forces with clear physical interpretations: they are the specific affinities for node escaping, edge exchange, and cycle completion. Practically, connecting the forces directly to the transition rates makes the abstract concept of a "fixed force constraint" physically realizable. This ultimately allows us to tackle explicit force constraints and evaluate network responses to kinetic rate or energetic perturbations.

B. General Response Relations and Symmetries

Because the Caliber $\mathfrak{c}(\mathfrak{F})$ is the scaled cumulant generating function, its derivatives directly yield the fluctuation-response relations. Specifically, the sensitivity of an average observable to its conjugate force evaluates to their asymptotic covariance:

$$\frac{\partial \bar{x}_\alpha}{\partial \mathfrak{F}_\beta} = \lim_{t\to\infty} t\, \mathrm{Cov}(x_\alpha, x_\beta) = \mathbf{C}_{\alpha\beta}, \tag{S12}$$

when *all other forces are held fixed*. Here, the indices α and β run through the path-dependent, stochastic observables $\mathbf{x} = (f_n, \phi_{ij}, \psi_c)$ defined in the previous section.

However, tuning forces while clamping other forces is not the same as tuning rates while fixing other rates. To map the fundamental force-response relations to the rate-response behaviors, we evaluate the sensitivity using the chain rule:

$$k_{ij} \frac{\partial \bar{x}_\alpha}{\partial k_{ij}} = \sum_\beta \frac{\partial \bar{x}_\alpha}{\partial \mathfrak{F}_\beta} k_{ij} \frac{\partial \mathfrak{F}_\beta}{\partial k_{ij}} = \sum_\beta \mathbf{C}_{\alpha\beta} \mathbf{A}_{(ij),\beta}. \tag{S13}$$

This naturally introduces the sparse Jacobian matrix $\mathbf{A}$, defined as:

$$\mathbf{A}_{(ij),\beta} \equiv k_{ij} \frac{\partial \mathfrak{F}_\beta}{\partial k_{ij}} = \frac{\partial \mathfrak{F}_\beta}{\partial \ln k_{ij}}. \tag{S14}$$

Based on the explicit force expressions (Eqs. S9-S11), the structure of $\mathbf{A}$ strictly encodes the local node-edge-cycle topology of the network, which will be useful in identifying response symmetries valid for any network topology.

To evaluate this rate sensitivity $k_{ij}\partial_{k_{ij}}\bar{x}_\alpha$, we parse the covariance matrix $\mathbf{C}_{\alpha\beta}$ using the intrinsic stochasticity of the system [14]. In a Markov jump process, the independent Poissonian noise source on each transition edge is defined as $\lambda_{ij} = (N_{ij} - T_i k_{ij})/t$. Because the raw counts N and T can be linearly spanned by the fundamental observables $\mathbf{X}$, the noise sources $\boldsymbol{\lambda}$ can be asymptotically expanded as a linear combination of the empirical rates $\mathbf{x} = \mathbf{X}/t$. It turns out this linear mapping is governed by the exact same Jacobian matrix $\mathbf{A}$ [14]:

$$\boldsymbol{\lambda} \sim \mathbf{A}\mathbf{x} - \nabla_{\ln \mathbf{k}} \mathfrak{c}. \tag{S15}$$

The term $\nabla_{\ln \mathbf{k}}\mathfrak{c}$ is simply a deterministic shift. Because the Caliber equates to the escape rate of the reference node m up to a constant (i.e., $\mathfrak{c} = \sum_{j(\neq m)}(k_{mj} - 1)$), the derivative $k_{uv}\partial_{k_{uv}}\mathfrak{c}$ is non-zero (yielding k_{mj}) only when the perturbed edge originates from the reference node ($u = m, v = j$).

Inverting this linear mapping yields $\mathbf{x} \sim \mathbf{A}^{-1}(\boldsymbol{\lambda} + \nabla_{\ln \mathbf{k}}\mathfrak{c})$. In the long-time limit, the independent noise sources possess a diagonal covariance: $\lim_{t\to\infty} t\, \mathrm{Cov}(\lambda_{ij}, \lambda_{ab}) = \pi_i k_{ij} \delta_{ia} \delta_{jb}$. Consequently, the covariance matrix of the observables is completely spanned by the independent edge noises via the inverse Jacobian:

$$\mathbf{C}_{\alpha\beta} = \sum_{ab} [\mathbf{A}^{-1}]_{\alpha,(ab)} [\mathbf{A}^{-1}]_{\beta,(ab)} (\pi_a k_{ab}). \tag{S16}$$

Substituting this covariance decomposition back into our chain rule expression yields:

$$k_{ij}\frac{\partial \bar{x}_\alpha}{\partial k_{ij}} = \sum_\beta \sum_{ab} [\mathbf{A}^{-1}]_{\alpha,(ab)} [\mathbf{A}^{-1}]_{\beta,(ab)} (\pi_a k_{ab}) \mathbf{A}_{(ij),\beta}. \tag{S17}$$

Because $\sum_\beta [\mathbf{A}^{-1}]_{\beta,(ab)} \mathbf{A}_{(ij),\beta} = \delta_{(ij),(ab)}$, the summation over ab collapses. This algebraic simplification perfectly cancels out one of the inverse matrices, yielding the **Response-Inverse-Matrix (RIM) relation** (first derived in [14]):

$$k_{ij}\frac{\partial \bar{x}_\alpha}{\partial k_{ij}} = p_{ij} [\mathbf{A}^{-1}]_{\alpha,(ij)}. \tag{S18}$$

The algebraic identity $\mathbf{A}^{-1}\mathbf{A} = \mathbf{I}$ generates a set of universal response symmetries. Multiplying a row α of $\mathbf{A}^{-1}$ (corresponding to an observable $\bar{x}_\alpha$) by a column β of $\mathbf{A}$ (corresponding to the conjugate force $\mathfrak{F}_\beta$) yields $\sum_{ij} [\mathbf{A}^{-1}]_{\alpha,(ij)} \mathbf{A}_{(ij),\beta} = \delta_{\alpha,\beta}$. Substituting the RIM relation translates these abstract matrix identities into three strict physical constraints on rate sensitivities [14]:

1. **Node Escaping Symmetry** (from column $\beta = f_n$ corresponding to $\mathfrak{F}_{\text{node},n}$):

$$\sum_{l(\neq m)} \frac{k_{ml}}{\pi_m} \frac{\partial \bar{x}_\alpha}{\partial k_{ml}} - \sum_{j(\neq n)} \frac{k_{nj}}{\pi_n} \frac{\partial \bar{x}_\alpha}{\partial k_{nj}} = \delta_{\bar{x}_\alpha, \pi_n}. \tag{S19}$$

2. **Edge Reciprocity** (from column $\beta = \phi_{ij}$ corresponding to $\mathfrak{F}_{\text{edge},ij}$):

$$\frac{1}{\pi_i}\frac{\partial \bar{x}_\alpha}{\partial k_{ij}} + \frac{1}{\pi_j}\frac{\partial \bar{x}_\alpha}{\partial k_{ji}} = 2\delta_{\bar{x}_\alpha, \tau_{ij}}. \tag{S20}$$

3. **Cycle Symmetry** (from column $\beta = \psi_c$ corresponding to $\mathfrak{F}_{\text{cycle},c}$):

$$\sum_{(ij)\in c^+} \frac{1}{\pi_i}\frac{\partial \bar{x}_\alpha}{\partial k_{ij}} - \sum_{(ji)\in c^-} \frac{1}{\pi_j}\frac{\partial \bar{x}_\alpha}{\partial k_{ji}} = 2\delta_{\bar{x}_\alpha, J_c}. \tag{S21}$$

These fundamental topological symmetries dictate the network's steady-state response to specific classes of kinetic parameter perturbations. In the Arrhenius kinetic parameterization [28], $k_{ij} \propto \exp[-(B_{ij} - E_i)]\exp(F_{ij}/2)$. This parameterization naturally classifies perturbations into state energy (E), kinetic barrier (B), and driving force (F) tuning. We explicitly evaluate the responses to state energy and kinetic barrier tuning below as these are the tunable knobs for molecular machines under fixed cycle forces.

1. Node Energy as an Isotropic Scaler

Perturbing the energy E_n of state n modifies all outgoing transition rates from that state proportionally. The perturbation operator is $\partial_{E_n} \equiv \sum_{j(\neq n)} k_{nj}\partial_{k_{nj}}$. Substituting this operator directly into the Node Escaping Symmetry (Eq. S19) yields:

$$\frac{1}{\pi_m}\frac{\partial \bar{x}_\alpha}{\partial E_m} - \frac{1}{\pi_n}\frac{\partial \bar{x}_\alpha}{\partial E_n} = \delta_{\bar{x}_\alpha, \pi_n}. \tag{S22}$$

For any flow observable distinct from probabilities (e.g., $\bar{x}_\alpha \in \{\tau_{ab}, J_c\}$), the Kronecker delta is zero. This requires that the normalized sensitivity $\frac{1}{\pi_n}\partial_{E_n}\bar{x}_\alpha$ is a global constant identical across all nodes in the network. Because steady-state fluxes and traffics possess physical units of inverse time, they scale linearly with a uniform, global shift in all node energies (which homogeneously scales all transition rates). By Euler's homogeneous function theorem of degree 1 [14], summing over all node perturbations yields $\sum_n \partial_{E_n}\bar{x}_\alpha = \bar{x}_\alpha$. Therefore, this network-wide constant must be $\bar{x}_\alpha$ itself, resulting in:

$$\frac{\partial \bar{x}_\alpha}{\partial E_n} = \pi_n \bar{x}_\alpha. \tag{S23}$$

This shows that node energies act purely as isotropic scalers. They scale the global magnitude of all network flows proportionally but cannot alter flux ratios.

2. *Kinetic Barrier as a Topological Router*

Perturbing the symmetric kinetic barrier B_{ij} on an edge modifies both the forward and backward rates equally and oppositely in logarithmic space. The operator is $\partial_{B_{ij}} \equiv -k_{ij}\partial_{k_{ij}} - k_{ji}\partial_{k_{ji}}$. Applying the Edge Reciprocity symmetry (Eq. S20), we substitute $k_{ji}\partial_{k_{ji}}\bar{x}_\alpha = 2\pi_j k_{ji}\delta_{\bar{x}_\alpha,\tau_{ij}} - \frac{\pi_j k_{ji}}{\pi_i}\partial_{k_{ij}}\bar{x}_\alpha$ directly into the barrier operator. This yields:

$$\begin{aligned}\frac{\partial \bar{x}_\alpha}{\partial B_{ij}} &= -k_{ij}\frac{\partial \bar{x}_\alpha}{\partial k_{ij}} - \left(2\pi_j k_{ji}\delta_{\bar{x}_\alpha,\tau_{ij}} - \frac{\pi_j k_{ji}}{\pi_i}\frac{\partial \bar{x}_\alpha}{\partial k_{ij}}\right) \\ &= -\frac{1}{\pi_i}\underbrace{(\pi_i k_{ij} - \pi_j k_{ji})}_{J_{ij}}\frac{\partial \bar{x}_\alpha}{\partial k_{ij}} - 2p_{ji}\delta_{\bar{x}_\alpha,\tau_{ij}}. \end{aligned} \tag{S24}$$

This response formula branches into two distinct cases depending on the target observable:

1. **For the local traffic ($\bar{x}_\alpha = \tau_{ij}$):** The Kronecker delta is 1, yielding:

$$\frac{\partial \tau_{ij}}{\partial B_{ij}} = -\frac{J_{ij}}{\pi_i}\frac{\partial \tau_{ij}}{\partial k_{ij}} - 2p_{ji}. \tag{S25}$$

   Even if the edge is detailed-balanced ($J_{ij} = 0$), raising the barrier still strictly reduces the local kinetic activity (traffic) by $-2p_{ji}$.

2. **For all other observables ($\bar{x}_\alpha \neq \tau_{ij}$):** The Kronecker delta vanishes, yielding:

$$\frac{\partial \bar{x}_\alpha}{\partial B_{ij}} = -\frac{J_{ij}}{\pi_i}\frac{\partial \bar{x}_\alpha}{\partial k_{ij}} = -J_{ij}[\mathbf{A}^{-1}]_{\alpha,(ij)}. \tag{S26}$$

   If the perturbed edge carries a zero net flux ($J_{ij} = 0$), the steady-state response $\partial_{B_{ij}}\bar{x}_\alpha$ is exactly zero. This explicitly establishes kinetic barriers as the exclusive routers of the network: a barrier perturbation can only influence the global distribution or flux if it is placed on an edge carrying a non-zero nonequilibrium net flux.

We remark that the Arrhenius energetic perturbations (E, B) discussed here are fundamentally distinct from tuning the intrinsic CFT forces $(\mathfrak{F}_{\text{node}}, \mathfrak{F}_{\text{edge}})$. Modifying a node energy E or a kinetic barrier B alters multiple transition rates simultaneously. In the CFT coordinate space, this corresponds to shifting several node and edge forces concurrently (i.e., moving vertically within the phase space where cycle forces are held constant). Nevertheless, their resulting responses are elegantly constrained by the topological symmetries derived via the Jacobian matrix. The theories about response relations presented up to this point—including the Jacobian mapping $\mathbf{A}$, the RIM relation, and the universal response symmetries—were a recapitulation of Ref. [14]. In later sections, we will utilize these established tools and symmetries to discover new operational principles for molecular machines.

II. EXAMPLE 1: F_1-ATPASE MOLECULAR ROTORS

A. The Gerritsma and Gaspard (2010) model and implementation details

To quantify the performance and operational bounds of the F_1-ATPase *in vitro*, we adopt the two-state, two-edge Markov network model parameterized by Gerritsma and Gaspard (2010) [15]. In this minimal model, the rotor transitions between an empty state (node 0) and a nucleotide-occupied state (node 1). These states are connected by two composite edges: the edge (T) representing ATP binding and unbinding, and the edge (D) representing ADP binding and release.

The four transition rates governing the network dynamics are defined as:

$$k_{\text{T}+}(\Gamma) = k_{+1}(\Gamma, \zeta)\,[\text{ATP}] \tag{S27}$$

$$k_{\text{T}-}(\Gamma) = k_{-1}(\Gamma, \zeta) \tag{S28}$$

$$k_{\text{D}-}(\Gamma) = k_{+2}(\Gamma, \zeta) \tag{S29}$$

$$k_{\text{D}+}(\Gamma) = k_{-2}(\Gamma, \zeta)\,[\text{ADP}][\text{P}_i] \tag{S30}$$

where Γ is the applied external torque and ζ is the viscous friction coefficient of the attached probe.

TABLE S1. **Coefficients for the F_1-ATPase transition rates.** Parameters are adapted from Gerritsma and Gaspard (2010) [15].

Coefficient	k_{+1} (ATP binding)	k_{+2} (Product release)	k_{-2} (Product binding)
$a^{(0)}$	-16.952	-5.973	-19.382
$a^{(1)}$ (pN^{-1} nm^{-1})	9.8×10^{-4}	1.7×10^{-4}	1.29×10^{-1}
$a^{(2)}$ (pN^{-2} nm^{-2})	5.8×10^{-4}	1.0×10^{-3}	2.8×10^{-4}
$b^{(0)}$	-16.352	-2.960	-18.338
$b^{(1)}$ (pN^{-1} nm^{-1})	-2.7×10^{-2}	-6.6×10^{-4}	5.9×10^{-3}
$b^{(2)}$ (pN^{-2} nm^{-2})	1.0×10^{-3}	3.6×10^{-4}	-2.1×10^{-4}

Based on their coarse-graining of a continuous Langevin model, the base rate constants k_ρ ($\rho \in \{+1, +2, -2\}$) incorporate the friction dependence via a non-Arrhenius crossover form:

$$k_\rho(\Gamma, \zeta) = \frac{1}{e^{a_\rho(\Gamma)} + \zeta e^{b_\rho(\Gamma)}} \tag{S31}$$

The torque-dependent coefficients are expanded up to the second order: $y_\rho(\Gamma) = y_\rho^{(0)} + y_\rho^{(1)}\Gamma + y_\rho^{(2)}\Gamma^2$ (for $y \in \{a, b\}$). The specific values for these coefficients, which ensure the model accurately reproduces the single-molecule experiments (see references within [15]), are summarized in Table S1.

The remaining ATP unbinding rate k_{-1} is determined by the thermodynamic local detailed balance (LDB) condition along the cycle:

$$k_{-1}(\Gamma, \zeta) = \frac{k_{+1}(\Gamma, \zeta) k_{+2}(\Gamma, \zeta)}{k_{-2}(\Gamma, \zeta)} \exp\left(\frac{\Delta G^0 - \frac{2\pi}{3}\Gamma}{k_B T}\right) \tag{S32}$$

where $\Delta G^0 = -50$ pN nm is the standard free energy of ATP hydrolysis.

Operational Regime and Simulation Parameters: To maintain the physical validity of the discrete-state coarse-graining and to avoid mechanical slipping—which would violate the tight-coupling assumption inherent to the 2-state network—we explicitly restrict our analysis to the applied torque range of $\Gamma \in [-30, 30]$ pN nm, as specified in the original study [15].

For all numerical evaluations and figures presented in this work, the thermal energy is set to $k_\mathrm{B}T = 4.1$ pN nm. The viscous friction coefficient ζ corresponds to a spherical probe of diameter $d = 160$ nm, evaluated as $\zeta = 2\pi \times 10^{-9} \times (d/2)^3 \times 4.75 \approx 0.0153$ pN·nm·s. Furthermore, we simulate the *in vitro* low-ATP conditions using the specific nucleotide concentrations: $[\mathrm{ATP}] = 0.49\ \mu$M, $[\mathrm{ADP}] = 100\ \mu$M, and $[\mathrm{P}_i] = 1$ mM. Under these exact boundary conditions, the total chemical driving force evaluates to $\Delta\mu = -\Delta G^0 + k_B T \ln([\mathrm{ATP}]/[\mathrm{ADP}][\mathrm{P}_i]) \approx 56.5$ pN nm. This explicitly dictates the thermodynamic stalling torque where the cycle flux vanishes, $\Gamma_\text{stall} = -\frac{3}{2\pi}\Delta\mu \approx -27.0$ pN nm. Finally, owing to this low ATP concentration, the ATP edge (T) acts as the dominant kinetic bottleneck throughout the entire functional cycle.

B. Equal Traffic processivity bound and inverse design

As established in the main text (Tool 1), Caliber Force Theory (CFT) permits switching between independent control variables, establishing the observable-force "canonical-like" coordinate $\mathbf{z} = (\boldsymbol{\pi}, \boldsymbol{\tau}, \mathfrak{F}_\text{cycle})$. This framework isolates the macroscopic free-energy drive (cycle force) from the internal kinetic layout. The associated dynamic free energy is the Legendre transform of the path entropy:

$$\mathcal{F}(\mathbf{z}) = \sum_c J_c\, \mathfrak{F}_{\text{cycle},c} + \mathfrak{s}_\text{path}, \tag{S33}$$

whose differentiation directly recovers the cycle flux: $J_c(\mathbf{z}) = \partial\mathcal{F}(\mathbf{z})/\partial\mathfrak{F}_{\text{cycle},c}$. For the two-edge F_1-ATPase network, this separation allows us to perform systematic inverse design by explicitly solving for the cycle flux J_c as a function of the traffic distribution.

The cycle force constraint dictates that the total environmental drive is exactly the sum of the internal edge affinities. In terms of the observable coordinates, this imposes the constraint:

$$\mathfrak{F}_\text{cycle} = \sum_{i=1}^{N} \operatorname{arctanh}\left(\frac{J_c}{\tau_i}\right). \tag{S34}$$

Before deriving the exact solution for the F_1-ATPase, we briefly recap the general *Equal Traffic Principle* (ETP) bound for an N-step cycle [13]. Because the function $\mathrm{arctanh}(J_c/\tau)$ is strictly convex with respect to τ (for $\tau > |J_c|$), applying Jensen's inequality to Eq. (S34) under a fixed total kinetic activity budget ($\tau_{\mathrm{tot}} = \sum \tau_i$) yields:

$$\frac{\mathfrak{F}_{\mathrm{cycle}}}{N} = \frac{1}{N}\sum_{i=1}^{N} \mathrm{arctanh}\left(\frac{J_c}{\tau_i}\right) \geq \mathrm{arctanh}\left(\frac{J_c}{\frac{1}{N}\tau_{\mathrm{tot}}}\right). \tag{S35}$$

Rearranging this inequality establishes the upper bound on the machine's processivity [13]:

$$\frac{J_c}{\tau_{\mathrm{tot}}} \leq \frac{1}{N}\tanh\left(\frac{\mathfrak{F}_{\mathrm{cycle}}}{N}\right) \tag{S36}$$

which saturates at equal traffic distributed across the cycle.

Exact Analytical Solution ($N = 2$): For the 2-edge F_1-ATPase network, we can solve the constraint exactly. Using the traffic fractions $\tau_{\mathrm{T}} = \rho\tau_{\mathrm{tot}}$ and $\tau_{\mathrm{D}} = (1-\rho)\tau_{\mathrm{tot}}$, where $\rho \in (0,1)$ is the traffic distribution ratio, Eq. (S34) becomes $\mathfrak{F}_{\mathrm{cycle}} = \mathrm{arctanh}(J_c/\tau_{\mathrm{T}}) + \mathrm{arctanh}(J_c/\tau_{\mathrm{D}})$. Applying the hyperbolic tangent addition formula, $\mathrm{arctanh}(a) + \mathrm{arctanh}(b) = \mathrm{arctanh}[(a+b)/(1+ab)]$, we rewrite this as:

$$\tanh(\mathfrak{F}_{\mathrm{cycle}}) = \frac{\frac{J_c}{\tau_{\mathrm{T}}} + \frac{J_c}{\tau_{\mathrm{D}}}}{1 + \frac{J_c^2}{\tau_{\mathrm{T}}\tau_{\mathrm{D}}}} = \frac{J_c(\tau_{\mathrm{T}} + \tau_{\mathrm{D}})\tau_{\mathrm{T}}\tau_{\mathrm{D}}}{\tau_{\mathrm{T}}\tau_{\mathrm{D}}(\tau_{\mathrm{T}}\tau_{\mathrm{D}} + J_c^2)} = \frac{J_c\tau_{\mathrm{tot}}}{\tau_{\mathrm{T}}\tau_{\mathrm{D}} + J_c^2}. \tag{S37}$$

Rearranging this yields a quadratic equation for the cycle flux J_c:

$$J_c^2 - \left[\tau_{\mathrm{tot}} \coth(\mathfrak{F}_{\mathrm{cycle}})\right] J_c + \tau_{\mathrm{T}}\tau_{\mathrm{D}} = 0. \tag{S38}$$

Because the physical net flux cannot exceed the symmetric traffic on any edge ($|J_c| \leq \min(\tau_{\mathrm{T}}, \tau_{\mathrm{D}})$), the physically valid root is strictly the smaller one. Substituting $\tau_{\mathrm{T}}\tau_{\mathrm{D}} = \rho(1-\rho)\tau_{\mathrm{tot}}^2$, we obtain the exact analytical mapping from the mixed coordinates to the cycle flux:

$$J_c(\rho, \tau_{\mathrm{tot}}, \mathfrak{F}_{\mathrm{cycle}}) = \frac{\tau_{\mathrm{tot}}}{2}\left[\coth(\mathfrak{F}_{\mathrm{cycle}}) - \sqrt{\coth^2(\mathfrak{F}_{\mathrm{cycle}}) - 4\rho(1-\rho)}\right]. \tag{S39}$$

From a formal CFT perspective, obtaining this explicit algebraic solution is mathematically equivalent to computing the exact derivative of the free energy potential, $J_c = \partial\mathcal{F}/\partial\mathfrak{F}_{\mathrm{cycle}}$.

Processivity Bound (ETP): The analytical solution Eq. (S39) makes the Equal Traffic Principle (ETP) strictly evident. For a fixed total kinetic activity τ_{tot} and fixed driving force $\mathfrak{F}_{\mathrm{cycle}}$, the flux J_c is maximized when the term inside the square root is minimized. This occurs exactly at the symmetric vertex $\rho = 1/2$ (i.e., $\tau_{\mathrm{T}} = \tau_{\mathrm{D}} = \tau_{\mathrm{tot}}/2$). At this ETP optimum, the term $4\rho(1-\rho) = 1$, and utilizing the identity $\coth^2(x) - 1 = \mathrm{csch}^2(x)$, the maximum achievable flux simplifies to:

$$\max(J_c) = \frac{\tau_{\mathrm{tot}}}{2}\left[\frac{\cosh(\mathfrak{F}_{\mathrm{cycle}}) - 1}{\sinh(\mathfrak{F}_{\mathrm{cycle}})}\right]. \tag{S40}$$

Applying the half-angle identities $\cosh(x) - 1 = 2\sinh^2(x/2)$ and $\sinh(x) = 2\sinh(x/2)\cosh(x/2)$, the processivity bound rigorously simplifies to:

$$\max\left(\frac{J_c}{\tau_{\mathrm{tot}}}\right) = \frac{1}{2}\tanh\left(\frac{\mathfrak{F}_{\mathrm{cycle}}}{2}\right). \tag{S41}$$

This proves the exact processivity bound, perfectly matching the $N = 2$ case of the general inequality (Eq. S36).

Rate Reconstruction and π-Degeneracy: Once J_c is computed via Eq. (S39) for any chosen traffic distribution ρ, the complete set of transition rates can be unambiguously reconstructed for any arbitrary state distribution $\pi_0 \in (0,1)$ (with $\pi_1 = 1 - \pi_0$). Utilizing the fundamental relations $k_{ij} = [\tau_{ij} + J_{ij}(J_c)]/(2\pi_i)$, the inverse design mapping is explicitly given by:

$$k_{\mathrm{T}+} = \frac{\rho\tau_{\mathrm{tot}} + J_c}{2\pi_0} \qquad k_{\mathrm{T}-} = \frac{\rho\tau_{\mathrm{tot}} - J_c}{2\pi_1}$$
$$k_{\mathrm{D}-} = \frac{(1-\rho)\tau_{\mathrm{tot}} + J_c}{2\pi_1} \qquad k_{\mathrm{D}+} = \frac{(1-\rho)\tau_{\mathrm{tot}} - J_c}{2\pi_0} \tag{S42}$$

This explicit reconstruction illuminates the $\boldsymbol{\pi}$-degeneracy in the inverse design discussed in the main text.

Cycle Symmetry and the Sum Theorem: We start from the universal Cycle Symmetry (Eq. S21) evaluated for the cycle flux $\bar{x}_\alpha = J_c$:

$$\sum_{(ij)\in c^+} \frac{1}{\pi_i}\frac{\partial J_c}{\partial k_{ij}} - \sum_{(ji)\in c^-} \frac{1}{\pi_j}\frac{\partial J_c}{\partial k_{ji}} = 2. \tag{S43}$$

The kinetic barrier perturbation operator on an edge ij modifies both forward and backward rates equally in logarithmic space: $\partial_{B_{ij}} \equiv -k_{ij}\partial_{k_{ij}} - k_{ji}\partial_{k_{ji}}$. By utilizing the Edge Reciprocity symmetry (Eq. S20), $\frac{1}{\pi_i}\partial_{k_{ij}} J_c = -\frac{1}{\pi_j}\partial_{k_{ji}} J_c$, the terms in the cycle symmetry summation simplify, and the barrier operator maps to the rate sensitivity via the local net flux J_{ij}:

$$\frac{\partial J_c}{\partial B_{ij}} = -\frac{J_{ij}}{\pi_i}\frac{\partial J_c}{\partial k_{ij}}. \tag{S44}$$

Substituting this mapping back into the Cycle Symmetry establishes a generic sum rule over kinetic barriers for any directed cycle:

$$\sum_{(ij)\in c} \frac{J_c}{J_{ij}}\frac{\partial \ln J_c}{\partial B_{ij}} = -1. \tag{S45}$$

This analytical relation provides a universal constraint linking local barrier perturbations to macroscopic cycle fluxes.

For a unicircular process like the F_1-ATPase model, probability conservation ensures the net flux along every edge is identical to the overall cycle flux ($J_\mathrm{T} = J_\mathrm{D} = J_c$). Consequently, the coefficients J_c/J_{ij} strictly evaluate to 1, collapsing the relation into a direct summation over the cycle's barriers:

$$\frac{\partial \ln J_c}{\partial B_\mathrm{T}} + \frac{\partial \ln J_c}{\partial B_\mathrm{D}} = -1. \tag{S46}$$

This uniform summation is readily verified by reading the analytical sensitivities directly from the cycle flux row (row 4) of the inverse Jacobian matrix $\mathbf{A}^{-1}$ derived above:

$$\left(-\frac{\Sigma_\mathrm{D}}{\Sigma}\right) + \left(-\frac{\Sigma_\mathrm{T}}{\Sigma}\right) = -\frac{\Sigma_\mathrm{D} + \Sigma_\mathrm{T}}{\Sigma} = -1. \tag{S47}$$

This explicitly validates the analytical inversion of the Jacobian and confirms its adherence to the network's topological constraints.

While this uniform summation $\sum \partial_B \ln J_c = -1$ serves as the stochastic network analog to the Flux Control Summation Theorem in Metabolic Control Analysis, it holds exclusively for unicircular processes. In multicyclic networks, the localized net fluxes J_{ij} diverge from the target cycle flux J_c, introducing edge-specific weighting coefficients (J_c/J_{ij}) into the barrier sum rule. Therefore, the foundational Cycle Symmetry constraint (Eq. S21) remains the more general and fundamental topological law.

C. Response Relations and Schur Complement Inversion

To map the topological routing responses of the F_1-ATPase, we evaluate its Jacobian matrix $\mathbf{A}$ and its inverse $\mathbf{A}^{-1}$ [14]. Recall from Section I.B that the Jacobian matrix is formally defined as $\mathbf{A}_{(ij),\alpha} \equiv k_{ij}\frac{\partial \mathfrak{F}_\alpha}{\partial k_{ij}}$, which links the microscopic rate perturbations to the macroscopic force responses. By choosing the rate representations $\mathbf{k} = (k_{\mathrm{T}+}, k_{\mathrm{D}-}, k_{\mathrm{T}-}, k_{\mathrm{D}+})$ and their corresponding conjugate forces $\mathfrak{F} = (\mathfrak{F}_\mathrm{edge,T}, \mathfrak{F}_\mathrm{edge,D}, \mathfrak{F}_\mathrm{node,1}, \mathfrak{F}_\mathrm{cycle})$, the analytical Jacobian matrix evaluates to:

$$\mathbf{A} = \begin{bmatrix} 1/2 & 0 & k_{\mathrm{T}+} & 1/2 \\ 0 & 1/2 & -k_{\mathrm{D}-} & 1/2 \\ 1/2 & 0 & -k_{\mathrm{T}-} & -1/2 \\ 0 & 1/2 & k_{\mathrm{D}+} & -1/2 \end{bmatrix}. \tag{S48}$$

Because of the network's local-global topological constraints, this sparse matrix possesses a highly symmetric block structure.

To invert this analytically, we partition the matrix into four 2×2 blocks: $\mathbf{A} = \begin{bmatrix} \mathbf{P} & \mathbf{Q} \\ \mathbf{R} & \mathbf{S} \end{bmatrix}$. Because the upper-left block $\mathbf{P}$ and the lower-left block $\mathbf{R}$ are simply diagonal matrices ($\mathbf{P} = \mathbf{R} = \frac{1}{2}\mathbf{I}$), their products simplify immensely: $\mathbf{P}^{-1} = 2\mathbf{I}$ and $\mathbf{R}\mathbf{P}^{-1} = \mathbf{I}$. Using the Schur complement $\mathbf{M} = \mathbf{S} - \mathbf{R}\mathbf{P}^{-1}\mathbf{Q} = \mathbf{S} - \mathbf{Q}$, we find:

$$\mathbf{M} = \begin{bmatrix} -k_{\mathrm{T}-} & -1/2 \\ k_{\mathrm{D}+} & -1/2 \end{bmatrix} - \begin{bmatrix} k_{\mathrm{T}+} & 1/2 \\ -k_{\mathrm{D}-} & 1/2 \end{bmatrix} = \begin{bmatrix} -\Sigma_{\mathrm{T}} & -1 \\ \Sigma_{\mathrm{D}} & -1 \end{bmatrix}, \tag{S49}$$

where $\Sigma_{\mathrm{X}} = k_{\mathrm{X}+} + k_{\mathrm{X}-}$ denotes the sum of rates on edge X. The determinant of the Schur complement is exactly the sum of all rates: $\det(\mathbf{M}) = \Sigma_{\mathrm{T}} + \Sigma_{\mathrm{D}} = \Sigma_{\mathrm{rates}}$.

Inverting $\mathbf{M}$ and applying the standard block matrix inversion formula yields the exact analytical inverse:

$$\mathbf{A}^{-1} = \begin{bmatrix} \mathbf{P}^{-1} + \mathbf{P}^{-1}\mathbf{Q}\mathbf{M}^{-1} & -\mathbf{P}^{-1}\mathbf{Q}\mathbf{M}^{-1} \\ -\mathbf{M}^{-1} & \mathbf{M}^{-1} \end{bmatrix} \tag{S50}$$

$$= \frac{1}{\Sigma_{\mathrm{rates}}} \begin{bmatrix} \Sigma_{\mathrm{D}} + 2k_{\mathrm{T}-} & \Delta_{\mathrm{T}} & \Sigma_{\mathrm{D}} + 2k_{\mathrm{T}+} & -\Delta_{\mathrm{T}} \\ -\Delta_{\mathrm{D}} & \Sigma_{\mathrm{T}} + 2k_{\mathrm{D}+} & \Delta_{\mathrm{D}} & \Sigma_{\mathrm{T}} + 2k_{\mathrm{D}-} \\ 1 & -1 & -1 & 1 \\ \Sigma_{\mathrm{D}} & \Sigma_{\mathrm{T}} & -\Sigma_{\mathrm{D}} & -\Sigma_{\mathrm{T}} \end{bmatrix}, \tag{S51}$$

where $\Delta_{\mathrm{X}} = k_{\mathrm{X}+} - k_{\mathrm{X}-}$. For the average observables $\bar{\mathbf{x}} = (\tau_{\mathrm{T}}, \tau_{\mathrm{D}}, \pi_1, J_c)$, this inverse matrix directly yields the exact rate sensitivities via the RIM relation (Eq. S18), $\partial_{k_{ij}} \bar{x}_\alpha = \pi_i [\mathbf{A}^{-1}]_{\alpha,(ij)}$, providing the explicit basis for the barrier sensitivity results discussed in the main text.

III. EXAMPLE 2: KINETIC PROOFREADING AND THE PERFORMANCE ENVELOPE

A. Construction of mixed coordinates and rate mapping

To evaluate the operational space of kinetic proofreading (KP) networks, we map the functional performance metrics to the transition rates using the Caliber Force Theory (CFT) mixed coordinate system. We model both the T7 DNA polymerase and the *E. coli* ribosome using a 5-state network: the empty enzyme (E), the initial cognate/non-cognate bound states (ER, EW), and the activated/catalytic states ($\mathrm{ER}^*, \mathrm{EW}^*$).

As illustrated in Fig. S1, to construct a linearly independent basis for net fluxes, we select a spanning tree consisting of the backbone transitions: $\mathrm{E} \rightleftharpoons \mathrm{ER} \rightleftharpoons \mathrm{ER}^*$ and $\mathrm{E} \rightleftharpoons \mathrm{EW} \rightleftharpoons \mathrm{EW}^*$. The four chords completing the fundamental cycles correspond to the irreversible product release and proofreading discard transitions. Based on the distinct topologies of the two machines, the fundamental cycle fluxes are identified as:

1. J_{R}: Cognate incorporation flux (chord ER $\to$ E for polymerase; $\mathrm{ER}^* \to$ E for ribosome).
2. J'_{R}: Cognate proofreading discard flux (chord $\mathrm{ER}^* \to$ E for both machines).
3. J_{W}: Non-cognate incorporation flux (chord EW $\to$ E for polymerase; $\mathrm{EW}^* \to$ E for ribosome).
4. J'_{W}: Non-cognate proofreading discard flux (chord $\mathrm{EW}^* \to$ E for both machines).

The performance metrics are algebraically defined by these fundamental fluxes:

$$S = J_{\mathrm{R}} + J_{\mathrm{W}}, \tag{S52}$$

$$\varepsilon = \log_{10}\left(\frac{J_{\mathrm{W}}}{J_{\mathrm{R}}}\right), \tag{S53}$$

$$C = S\Delta\mu_{\mathrm{p}} + (J'_{\mathrm{R}} + J'_{\mathrm{W}})\Delta\mu_{\mathrm{NTP}}, \tag{S54}$$

$$r' = \frac{J'_{\mathrm{W}}}{J'_{\mathrm{R}}}. \tag{S55}$$

Here, $\Delta\mu_{\mathrm{p}}$ is the free energy of bond formation and $\Delta\mu_{\mathrm{NTP}}$ is the free energy of NTP hydrolysis. Assuming the network operates in a functional regime where both incorporation fluxes are positive ($J_{\mathrm{R}} > 0, J_{\mathrm{W}} > 0$), we invert this system to explicitly solve

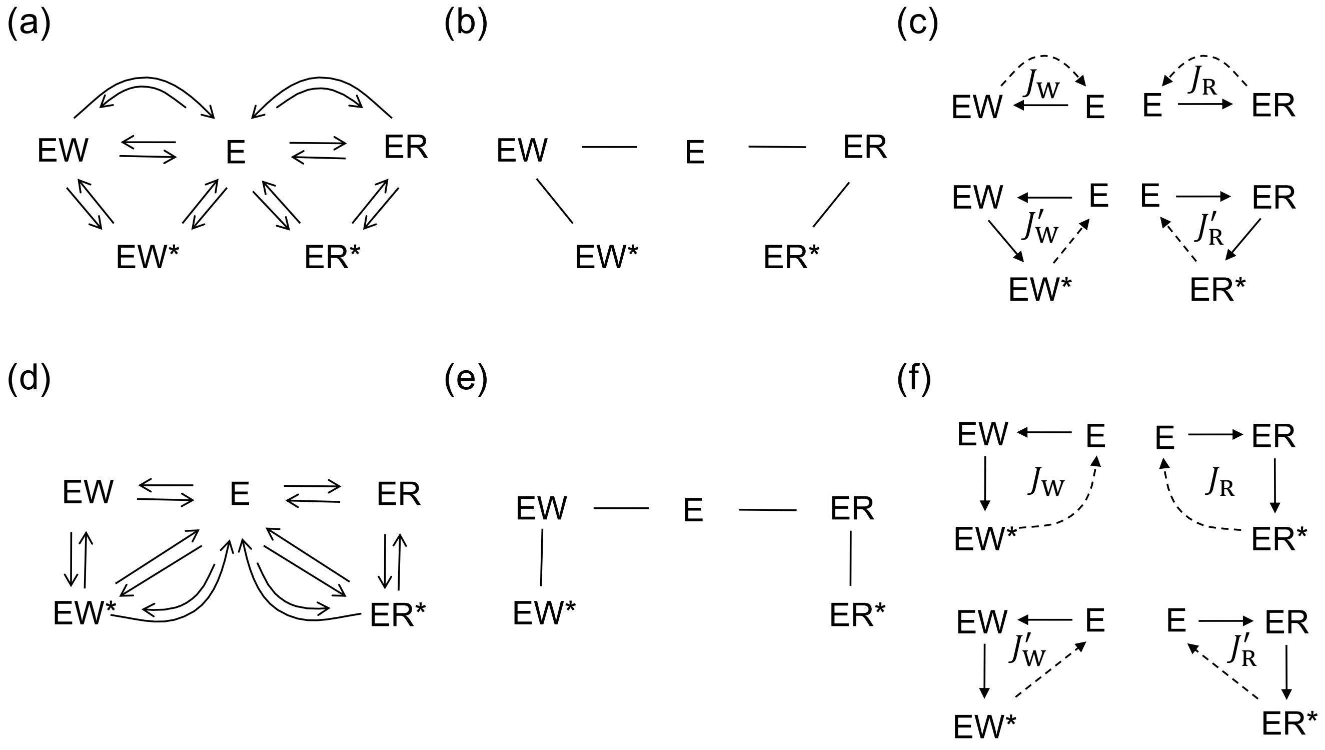

FIG. S1. **Network topologies, spanning trees, and fundamental cycles for the kinetic proofreading models.** (a) The 5-state network state space for the T7 DNA polymerase. (b) The chosen spanning tree (solid lines) consisting of the backbone transitions. (c) The fundamental cycles formed by adding the chord transitions (dashed arrows) and removing the redundant edges in the spanning tree. These chords define the cognate/non-cognate incorporation (J_R, J_W) and proofreading discard (J'_R, J'_W) fluxes. (d-f) The corresponding network state space, spanning tree, and fundamental cycles for the *E. coli* ribosome, highlighting the distinct topological origin of its productive incorporation transitions.

for the fundamental cycle fluxes as functions of the target coordinate (S, ε, C, r'):

$$J_\mathrm{R} = \frac{S}{1+10^\varepsilon}, \quad J_\mathrm{W} = \frac{S \cdot 10^\varepsilon}{1+10^\varepsilon}, \tag{S56}$$

$$J'_\mathrm{R} = \frac{C - S\Delta\mu_\mathrm{p}}{\Delta\mu_\mathrm{NTP}(1+r')}, \quad J'_\mathrm{W} = \frac{r'(C - S\Delta\mu_\mathrm{p})}{\Delta\mu_\mathrm{NTP}(1+r')}. \tag{S57}$$

These explicit relations are crucial for substituting the abstract cycle fluxes with tangible functional targets in our inverse design coordinate.

Coordinate Swap via Cycle Forces: The key to enabling inverse design is transforming the "microcanonical" coordinate $\bar{\mathbf{x}} = (\boldsymbol{\pi}, \boldsymbol{\tau}, \boldsymbol{J})$ into a canonical-like coordinate where the cycle forces $\mathfrak{F}_\mathrm{cycle}$ are fixed. For any fundamental cycle c, the cycle force is the sum of its edge affinities:

$$\mathfrak{F}_{\mathrm{cycle},c} = \operatorname{arctanh}\left(\frac{J_c}{\tau_c}\right) + \sum_{(ij)\in c\setminus\{c\}} \operatorname{arctanh}\left(\frac{J_{ij}}{\tau_{ij}}\right), \tag{S58}$$

where τ_c is the traffic exclusively on the defining chord. Because the arctanh function is strictly monotonic and invertible, we can analytically isolate τ_c:

$$\tau_c = J_c \coth\left(\mathfrak{F}_{\mathrm{cycle},c} - \sum_{(ij)\in c\setminus\{c\}} \operatorname{arctanh}\left(\frac{J_{ij}}{\tau_{ij}}\right)\right). \tag{S59}$$

This explicit substitution removes the four chord traffics from the set of independent variables. The remaining edge traffics along the spanning tree are denoted as $\boldsymbol{\tau}_\mathrm{rem}$. This establishes the exact analytical mapping into the mixed coordinate system

$(\boldsymbol{\pi}, \boldsymbol{\tau}_{\text{rem}}, \boldsymbol{J}, \mathfrak{F}_{\text{cycle}})$.

Furthermore, because the four cycle forces are explicitly constrained by the thermodynamic driving forces ($2\mathfrak{F}_{\text{cycle, R}} = 2\mathfrak{F}_{\text{cycle, W}} = \Delta\mu_{\text{p}}$ for the incorporation cycles, and $2\mathfrak{F}'_{\text{cycle, R}} = 2\mathfrak{F}'_{\text{cycle, W}} = \Delta\mu_{\text{NTP}}$ for the discard cycles), and the fundamental cycle fluxes $\boldsymbol{J}$ can be fully parameterized by the performance targets (S, ε, C, r') via Eqs. (S56)–(S57), we ultimately construct the inverse design coordinate: $(\boldsymbol{\pi}, \boldsymbol{\tau}_{\text{rem}}, S, \varepsilon, C, r', \mathfrak{F}_{\text{cycle}})$.

Systematic Rate Reconstruction: To translate an operational target point in this functional coordinate space back to the 16 microscopic transition rates, we execute the following sequence:

1. Evaluate the four fundamental cycle fluxes $J_c \in \{J_{\text{R}}, J_{\text{W}}, J'_{\text{R}}, J'_{\text{W}}\}$ from the target metrics (S, ε, C, r') using Eqs. (S56) and (S57).

2. Compute the local net flux J_{ij} on every edge by superimposing the fundamental cycle fluxes traversing it. For instance, the initial cognate binding edge E $\rightleftharpoons$ ER participates in both the incorporation and discard cycles, yielding $J_{\text{E}\to\text{ER}} = J_{\text{R}} + J'_{\text{R}}$. As another example mapped in Fig. S1, the activation edge ER $\rightleftharpoons$ ER* in the T7 DNA polymerase carries exclusively the proofreading discard flux ($J_{\text{ER}\to\text{ER}^*} = J'_{\text{R}}$), whereas the same edge in the *E. coli* ribosome carries both fluxes ($J_{\text{ER}\to\text{ER}^*} = J_{\text{R}} + J'_{\text{R}}$).

3. With all local fluxes J_{ij} determined, substitute the fixed cycle forces $\mathfrak{F}_{\text{cycle}}$ and the remaining tree traffics $\boldsymbol{\tau}_{\text{rem}}$ into Eq. (S59) to algebraically resolve the four dependent chord traffics τ_c.

4. Having completely specified all state probabilities π_i, edge traffics τ_{ij}, and local fluxes J_{ij}, the 16 exact microscopic transition rates are reconstructed via the fundamental CFT mapping:

$$k_{ij} = \frac{\tau_{ij} + J_{ij}}{2\pi_i}, \quad k_{ji} = \frac{\tau_{ij} - J_{ij}}{2\pi_j}. \tag{S60}$$

This sequence perfectly isolates the physical parameters, allowing us to explicitly design the microscopic transition rates that realize any mathematically feasible set of performance targets.

B. Traffic bounds and anti-proofreading regimes

The rate reconstruction in Eq. (S60) is physically bounded by the non-negativity of the one-way probability fluxes, $p_{ij} = (\tau_{ij} + J_{ij})/2 \geq 0$. This imposes the strict physical constraint $|J_{ij}| \leq \tau_{ij}$ on every edge.

Projecting the limit $J_{\text{E}\to\text{ER}} \leq \tau_{\text{E, ER}}$ into the functional coordinate space using Eqs. (S56) and (S57) yields the trade-off bound presented in the main text:

$$\underbrace{\frac{S}{1+10^{\varepsilon}}}_{J_R} + \underbrace{\frac{C - S\Delta\mu_{\text{p}}}{\Delta\mu_{\text{NTP}}(1+r')}}_{J'_R} \leq \tau_{\text{E, ER}}. \tag{S61}$$

This inequality demonstrates a kinetic traffic jam: the productive incorporation speed and the proofreading dissipation must compete for the shared finite bandwidth $\tau_{\text{E, ER}}$. Similarly, the flux limit on the non-cognate binding edge, $J_{\text{E}\to\text{EW}} \leq \tau_{\text{E, EW}}$, establishes a parallel boundary for the non-cognate pathway:

$$\underbrace{\frac{S \cdot 10^{\varepsilon}}{1+10^{\varepsilon}}}_{J_{\text{W}}} + \underbrace{\frac{r'(C - S\Delta\mu_{\text{p}})}{\Delta\mu_{\text{NTP}}(1+r')}}_{J'_{\text{W}}} \leq \tau_{\text{E, EW}}. \tag{S62}$$

Furthermore, the independent chord transitions impose strict boundaries on the individual discard fluxes: $|J'_{\text{R}}| \leq \tau_{\text{ER}^*,\text{E}}, \quad |J'_{\text{W}}| \leq \tau_{\text{EW}^*,\text{E}}$.

Anti-Proofreading Regime: The standard proofreading regime operates under positive NTP dissipation ($C \geq S\Delta\mu_{\text{p}}$), yielding $J'_{\text{R}}, J'_{\text{W}} \geq 0$. However, driving the energetic cost C below the incorporation baseline $S\Delta\mu_{\text{p}}$ forces the net discard fluxes to become negative. In this anti-proofreading regime, the network must run the discard cycles in reverse to synthesize NTP.

While Eq. (S62) represents the upper capacity bound, a highly negative non-cognate discard flux triggers the corresponding lower bound constraint:

$$J_\mathrm{W} + J'_\mathrm{W} \geq -\tau_\mathrm{E,\,EW}. \tag{S63}$$

When the reversed non-cognate discard flux strictly exceeds the available binding traffic bandwidth ($J'_\mathrm{W} < -\tau_\mathrm{E,\,EW}$, or equivalently $|J'_\mathrm{W}| > \tau_\mathrm{E,\,EW}$), we substitute J_W and $-|J'_\mathrm{W}|$ into the lower bound:

$$S\frac{10^\varepsilon}{1+10^\varepsilon} - |J'_\mathrm{W}| \geq -\tau_\mathrm{E,\,EW}. \tag{S64}$$

Multiplying both sides by $(1+10^\varepsilon)$ and isolating the 10^ε terms yields the intermediate step:

$$10^\varepsilon \left(S - |J'_\mathrm{W}| + \tau_\mathrm{E,\,EW}\right) \geq |J'_\mathrm{W}| - \tau_\mathrm{E,\,EW}. \tag{S65}$$

By substituting $-|J'_\mathrm{W}| = J'_\mathrm{W}$ back into the left-hand parenthesis, this algebraically reorganizes into a strict lower bound on the error rate:

$$10^\varepsilon \geq \frac{|J'_\mathrm{W}| - \tau_\mathrm{E,\,EW}}{S + J'_\mathrm{W} + \tau_\mathrm{E,\,EW}}. \tag{S66}$$

This establishes a minimum achievable error floor, revealing a topological constraint when the system is driven into regimes of negative proofreading dissipation.

C. Algorithmic details of the linear exploration

To navigate the functional interior without violating these physical limits, we inversely designed an optimization protocol (Main Text Fig. 5c, d) operating directly within our key coordinate $(\boldsymbol{\pi}, \boldsymbol{\tau}_\mathrm{rem}, S, \varepsilon, C, r', \mathfrak{F}_\mathrm{cycle})$. The algorithm explores the concurrent improvement of speed, accuracy, and cost as follows:

1. **Initialization:** The coordinate is initialized at the wild-type parameters $\mathbf{z}_\mathrm{WT}$ derived from Mallory *et al.* [19], explicitly listed in Table S2. The state probabilities $\boldsymbol{\pi}$, the spanning-tree traffics $\boldsymbol{\tau}_\mathrm{rem}$, and the discard ratio r' are clamped to their wild-type values.

2. **Linear Progression:** The target functional variables are driven along a linear sequence parameterized by λ:

$$S(\lambda) = S_\mathrm{WT} + \lambda\Delta S, \quad \varepsilon(\lambda) = \varepsilon_\mathrm{WT} - \lambda\Delta\varepsilon, \quad C(\lambda) = C_\mathrm{WT} - \lambda\Delta C. \tag{S67}$$

   For the explicit optimization trajectories demonstrated in the main text, the directional vector is set to $\Delta S = 3\times10^{-7}\ \mathrm{s}^{-1}$, $\Delta\varepsilon = -0.02$, and $\Delta C = -1.0\ k_\mathrm{B}T$.

3. **Traffic Relaxation Rule:** At each step λ, the required local fluxes $J_{ij}(\lambda)$ are evaluated. We continuously monitor the saturation ratio $|J_{ij}|/\tau_{ij}$ for all edges. If any edge collides with the physical boundary ($|J_{ij}|/\tau_{ij} \geq 1.0$), the local kinetic bandwidth is expanded by multiplying the traffic of that specific edge by 10 ($\tau_{ij} \to 10\tau_{ij}$). This allows the free-lunch optimization trajectory to proceed beyond the initial wild-type kinetic envelope.

4. **Rate Execution:** With the dynamically updated traffics and fluxes, the transition rates $k_{ij}(\lambda)$ are generated via the analytical mapping in Eq. (S60).

The abrupt jumps observed in the engineered transition rates (Main Text Fig. 5d) are the direct consequence of these traffic relaxation events triggering to prevent negative probability fluxes.

IV. EXAMPLE 3: ENZYME INHIBITION AND NON-INVERTIBLE NETWORKS

A. Caliber Force construction for non-invertible networks

In this section, we provide the construction of the Caliber Force Theory (CFT) framework for the noncompetitive inhibition model. This model is unique because it incorporates a non-invertible catalytic step, representing the chemical production of the enzyme. The Maximum Caliber framework maximizes the path entropy relative to a reference process. The resulting

Transition (s^{-1})	T7 DNA Polymerase Cognate (k_R)	Non-Cognate (k_W)	*E. coli* Ribosome Cognate (k_R)	Non-Cognate (k_W)
k_1 (E $\rightarrow$ EX)	250.0	2.0×10^{-3}	40.0	27.0
k_{-1} (EX $\rightarrow$ E)	1.0	1.0×10^{-5}	0.5	47.0
k_2 (EX $\rightarrow$ EX*)	0.2	2.3	25.0	1.2
k_{-2} (EX* $\rightarrow$ EX)	700.0	700.0	1.0×10^{-3}	1.0×10^{-3}
k_3 (Discard)	900.0	900.0	8.5×10^{-2}	0.6715
k_{-3} (Reverse Discard)	1.32×10^{-7} *	1.22×10^{-9} *	3.50×10^{-4} *	9.54×10^{-7} *
k_p (Incorporation)	250.0	1.2×10^{-2}	8.415	3.53×10^{-2}
k_{-p} (Reverse Incorp.)	3.20×10^{-7} *	1.23×10^{-11} *	8.60×10^{-5} *	1.24×10^{-10} *

TABLE S2. **Explicit wild-type transition rates for the kinetic proofreading models.** All 16 microscopic transition rates for the cognate (Right) and non-cognate (Wrong) pathways are explicitly listed to ensure theoretical reproducibility, adapted from Mallory *et al.* [19]. Rates marked with an asterisk (*) were computed to enforce local detailed balance. Specifically, the reverse proofreading discard rates were analytically constrained by the free energy of NTP hydrolysis ($\Delta\mu_{\text{NTP}} = 20.0\ k_B T$) via $k_{-3} = (k_1 k_2 k_3)/(k_{-1} k_{-2} e^{\Delta\mu_{\text{NTP}}})$. Similarly, the reverse incorporation rates were constrained by the bond formation free energy ($\Delta\mu_{\text{p}} = 26.0\ k_B T$). Due to the distinct topologies of the production cycles, $k_{-p} = (k_1 k_p)/(k_{-1} e^{\Delta\mu_{\text{p}}})$ for the T7 DNA polymerase, whereas $k_{-p} = (k_1 k_2 k_p)/(k_{-1} k_{-2} e^{\Delta\mu_{\text{p}}})$ for the *E. coli* ribosome.

parameterization of path probability is still an exponential family, and the conjugate coordinates still exist and are still converted by Legendre-Fenchel transforms.

1. Model Topology and Transition Matrix: We consider a four-state enzyme system: free enzyme E (node 0), enzyme-substrate complex ES (node 1), enzyme-inhibitor-substrate complex EIS (node 2), and enzyme-inhibitor complex EI (node 3). The network is connected by invertible binding transitions, but the production step is non-invertible: ES $\xrightarrow{k_{\text{cat}}}$ E ($1 \rightarrow 0$). The transition rate matrix $\mathbf{k}$ describes the rate of probability flow between these states:

$$\mathbf{k} = \begin{bmatrix} -\sum_i k_{0i} & k_{01} & 0 & k_{03} \\ k_{10} + k_{\text{cat}} & -\sum_j k_{1j} - k_{\text{cat}} & k_{12} & 0 \\ 0 & k_{21} & -\sum_l k_{2l} & k_{23} \\ k_{30} & 0 & k_{32} & -\sum_m k_{3m} \end{bmatrix}. \tag{S68}$$

This explicit matrix formulation serves as the mathematical basis for extracting the conjugate forces.

2. Observable Basis and Fundamental Cycles: To construct the linearly independent observable basis $\bar{\mathbf{x}}$, we select node 0 (E) as the reference node m, making π_1, π_2, π_3 the independent state probabilities. We then select a spanning tree covering the transitions $1 \rightleftharpoons 0 \rightleftharpoons 3 \rightleftharpoons 2$. The fundamental cycles are defined by adding the remaining chords. The reversible binding edge $1 \rightleftharpoons 2$ forms the invertible leak cycle ($0 \rightleftharpoons 3 \rightleftharpoons 2 \rightleftharpoons 1 \rightleftharpoons 0$), with its net flux denoted as $J_{\text{leak}} = J_{21}$. The non-invertible catalytic edge $1 \rightarrow 0$ acts as the defining chord for the production cycle ($0 \rightleftharpoons 1 \rightarrow 0$), carrying the flux J_{cat}. Because the catalytic step is non-invertible, its symmetric traffic τ_{cat} strictly equals its directed flux J_{cat} and is thus removed from the set of independent variables. The resulting observable coordinate vector is $\bar{\mathbf{x}} = (\tau_{01}, \tau_{03}, \tau_{12}, \tau_{23}, \pi_1, \pi_2, \pi_3, J_{\text{cat}}, J_{\text{leak}})$.

3. Tilted Matrix and Conjugate Forces: To identify the rate expressions of conjugate forces, we resort to the rate parameterization in Eq. (S8a), which requires first constructing the tilted matrix $\tilde{\mathbf{M}}$. The off-diagonal elements are tilted by the forces associated with each specific transition. The diagonal elements capture node constraints defined by the out-degree of each node in the reference "doldrum" process (e.g., node 1 connects to nodes 0 and 2 via reversible edges and node 0 via the catalytic edge, yielding an out-degree of 3). The largest eigenvalue of $\tilde{\mathbf{M}}$ is the Caliber $\mathfrak{c}$, which acts as the dynamical partition function:

$$\tilde{\mathbf{M}} = \begin{bmatrix} -2 & e^{\mathfrak{F}_{\text{edge},01}} & 0 & e^{\mathfrak{F}_{\text{edge},03}} \\ e^{\mathfrak{F}_{\text{edge},01}} + e^{\mathfrak{F}_{\text{cat}}} & \mathfrak{F}_{\text{node},1} - 3 & e^{\mathfrak{F}_{\text{edge},12} - \mathfrak{F}_{\text{leak}}} & 0 \\ 0 & e^{\mathfrak{F}_{\text{edge},12} + \mathfrak{F}_{\text{leak}}} & \mathfrak{F}_{\text{node},2} - 2 & e^{\mathfrak{F}_{\text{edge},23}} \\ e^{\mathfrak{F}_{\text{edge},03}} & 0 & e^{\mathfrak{F}_{\text{edge},23}} & \mathfrak{F}_{\text{node},3} - 2 \end{bmatrix}. \tag{S69}$$

The right eigenvector $\mathbf{v}$ associated with $\mathfrak{c}$ bridges the macroscopic forces back to the microscopic transition rates via $k_{ij} =$

$\tilde{M}_{ij} v_j / v_i$. The corresponding rate matrix $\mathbf{k}$ evaluates to:

$$\mathbf{k} = \begin{bmatrix} -2 - \mathfrak{c} & e^{\mathfrak{F}_{\text{edge},01}} \frac{v_1}{v_0} & 0 & e^{\mathfrak{F}_{\text{edge},03}} \frac{v_3}{v_0} \\ e^{\mathfrak{F}_{\text{edge},01}} \frac{v_0}{v_1} + e^{\mathfrak{F}_{\text{cat}}} \frac{v_0}{v_1} & \mathfrak{F}_{\text{node},1} - 3 - \mathfrak{c} & e^{\mathfrak{F}_{\text{edge},12} - \mathfrak{F}_{\text{leak}}} \frac{v_2}{v_1} & 0 \\ 0 & e^{\mathfrak{F}_{\text{edge},12} + \mathfrak{F}_{\text{leak}}} \frac{v_1}{v_2} & \mathfrak{F}_{\text{node},2} - 2 - \mathfrak{c} & e^{\mathfrak{F}_{\text{edge},23}} \frac{v_3}{v_2} \\ e^{\mathfrak{F}_{\text{edge},03}} \frac{v_0}{v_3} & 0 & e^{\mathfrak{F}_{\text{edge},23}} \frac{v_2}{v_3} & \mathfrak{F}_{\text{node},3} - 2 - \mathfrak{c} \end{bmatrix}. \tag{S70}$$

Evaluating the logarithmic ratios of these reconstructed rates along specific network structures completely cancels out the eigenvector components. To extract the catalytic cycle force, we evaluate k_{cat} and the reversible binding rates on the E $\rightleftharpoons$ ES edge. Since $k_{\text{cat}} = e^{\mathfrak{F}_{\text{cat}}} \frac{v_0}{v_1}$ and the symmetric binding rates satisfy $\frac{k_{01}}{k_{10}} = (\frac{v_1}{v_0})^2$, taking the logarithm yields $\ln k_{\text{cat}} + \frac{1}{2} \ln \frac{k_{01}}{k_{10}} = \mathfrak{F}_{\text{cat}} + \ln \frac{v_0}{v_1} + \ln \frac{v_1}{v_0} = \mathfrak{F}_{\text{cat}}$. Repeating this for the remaining structures yields the explicit algebraic mapping from rates to conjugate forces:

$$\mathfrak{F}_{\text{node},n} = \sum_i (k_{0i} - 1) - \sum_j (k_{nj} - 1), \tag{S71}$$

$$\mathfrak{F}_{\text{edge},ij} = \frac{1}{2} \ln(k_{ij} k_{ji}), \tag{S72}$$

$$\mathfrak{F}_{\text{leak}} = \frac{1}{2} \sum_{(ij) \in 0 \to 3 \to 2 \to 1 \to 0} \ln \frac{k_{ij}}{k_{ji}}, \tag{S73}$$

$$\mathfrak{F}_{\text{cat}} = \ln k_{\text{cat}} + \frac{1}{2} \ln \frac{k_{01}}{k_{10}}. \tag{S74}$$

This demonstrates that the inclusion of a non-invertible step only modifies the definition of the specific cycle force traversing it ($\mathfrak{F}_{\text{cat}}$), while preserving the standard force definitions for all nodes, edges, and invertible cycles ($\mathfrak{F}_{\text{leak}}$).

4. The Jacobian Matrix A: The Jacobian matrix $\mathbf{A}_{(ij),\alpha} = k_{ij} \partial \mathfrak{F}_\alpha / \partial k_{ij}$ serves as the dictionary mapping local rate perturbations to global force responses. We define the rate vector $\mathbf{k} = (k_{01}, k_{10}, k_{\text{cat}}, k_{03}, k_{30}, k_{12}, k_{21}, k_{23}, k_{32})^\mathsf{T}$ as the rows, and the conjugate force vector $\boldsymbol{\mathfrak{F}} = (\mathfrak{F}_{\text{edge},01}, \mathfrak{F}_{\text{edge},03}, \mathfrak{F}_{\text{edge},12}, \mathfrak{F}_{\text{edge},23}, \mathfrak{F}_{\text{node},1}, \mathfrak{F}_{\text{node},2}, \mathfrak{F}_{\text{node},3}, \mathfrak{F}_{\text{cat}}, \mathfrak{F}_{\text{leak}})^\mathsf{T}$ as the columns. The 9×9 Jacobian is analytically evaluated as:

$$\mathbf{A} = \begin{bmatrix} 1/2 & 0 & 0 & 0 & k_{01} & k_{01} & k_{01} & 1/2 & -1/2 \\ 1/2 & 0 & 0 & 0 & -k_{10} & 0 & 0 & -1/2 & 1/2 \\ 0 & 0 & 0 & 0 & -k_{\text{cat}} & 0 & 0 & 1 & 0 \\ 0 & 1/2 & 0 & 0 & k_{03} & k_{03} & k_{03} & 0 & 1/2 \\ 0 & 1/2 & 0 & 0 & 0 & 0 & -k_{30} & 0 & -1/2 \\ 0 & 0 & 1/2 & 0 & -k_{12} & 0 & 0 & 0 & -1/2 \\ 0 & 0 & 1/2 & 0 & 0 & -k_{21} & 0 & 0 & 1/2 \\ 0 & 0 & 0 & 1/2 & 0 & -k_{23} & 0 & 0 & -1/2 \\ 0 & 0 & 0 & 1/2 & 0 & 0 & -k_{32} & 0 & 1/2 \end{bmatrix}. \tag{S75}$$

The independent edge noise sources are defined as $\lambda_{ij} = (N_{ij} - T_i k_{ij})/t$, where N_{ij} are the transition counts and T_i are the dwell times. The empirical observable rates are $\mathbf{x} = (f_n, \phi_{ij}, \psi_c) = (T_n, \Phi_{ij}, \Psi_c)/t$. Because node probability normalization ($\sum_n f_n = 1$) and Kirchhoff's current law ($\sum_j N_{ij} - \sum_j N_{ji} \sim 0$) constrain the raw counts, we can algebraically substitute the counting variables N and T with the independent basis $\mathbf{x}$. While we omit the algebraic substitution here (detailed in Ref. [14]), this demonstrates that the linear mapping between the noise sources $\boldsymbol{\lambda}$ and the fluctuating observables $\mathbf{x}$ remains governed by this Jacobian: $\boldsymbol{\lambda} \sim \mathbf{A}\mathbf{x} - \nabla_{\ln \mathbf{k}} \mathfrak{c}$. Because this stochastic mapping holds, the derivation of the Response-Inverse-Matrix (RIM) relation ($\partial \bar{x}_\alpha / \partial \ln k_{ij} = \pi_i \mathbf{A}^{-1}_{\alpha,(ij)}$) provided in Eq. (S18) remains valid for networks with non-invertible transitions.

B. Explicit derivation of the Inhibitor Equal Traffic Principle

1. Flux Conservation and Force Balance: The inner loop E $\rightleftharpoons$ ES $\rightleftharpoons$ EIS $\rightleftharpoons$ EI $\rightleftharpoons$ E is futile with no net effect, requiring its cycle force to vanish ($\mathfrak{F}_{\text{leak}} = 0$). However, the irreversible catalytic sink at state 1 (ES $\to$ E) induces a steady-state compensatory backflow, J_{leak}.

Applying Kirchhoff's Current Law at each node dictates the flow routing:

1. At node 1 (ES): The net flux from node 0 (J_{01}) must satisfy $J_{01} = J_{\text{cat}} - J_{\text{leak}}$.

2. At the sideway nodes (2 and 3): The net fluxes J_{21}, J_{32}, J_{03} are equal to J_{leak}, which strictly implies $J_{12}, J_{23}, J_{30} = -J_{\text{leak}}$.

Denoting the edge affinity $\mathcal{A}_{ij} = \text{arctanh}(J_{ij}/\tau_{ij})$, the zero cycle force constraint $\sum_{(ij)\in\text{cycle}} \mathcal{A}_{ij} = 0$ along the loop $0 \to 1 \to 2 \to 3 \to 0$ is expressed as:

$$\text{arctanh}\left(\frac{J_{\text{cat}} - J_{\text{leak}}}{\tau_{01}}\right) - \left[\text{arctanh}\left(\frac{J_{\text{leak}}}{\tau_{12}}\right) + \text{arctanh}\left(\frac{J_{\text{leak}}}{\tau_{23}}\right) + \text{arctanh}\left(\frac{J_{\text{leak}}}{\tau_{30}}\right)\right] = 0. \tag{S76}$$

Let $\mathcal{A}_{\text{side}} = \sum_{k\in\{12,23,30\}} \text{arctanh}(J_{\text{leak}}/\tau_k)$. Applying the hyperbolic tangent to both sides of Eq. (S76) isolates the catalytic production:

$$\frac{J_{\text{cat}} - J_{\text{leak}}}{\tau_{01}} = \tanh(\mathcal{A}_{\text{side}}) \implies J_{\text{cat}} = J_{\text{leak}} + \tau_{01}\tanh(\mathcal{A}_{\text{side}}). \tag{S77}$$

This algebraic isolation of J_{cat} establishes the coordinate transformation. We start from the observable coordinate $(\boldsymbol{\pi}, \boldsymbol{\tau}, J_{\text{cat}}, J_{\text{leak}})$. By enforcing the thermodynamic constraint $\mathfrak{F}_{\text{leak}} = 0$, we substitute J_{cat} out of the independent variables, yielding the mixed coordinate $\mathbf{z} = (\boldsymbol{\pi}, \boldsymbol{\tau}, J_{\text{leak}}, \mathfrak{F}_{\text{leak}})$. As demonstrated in Example 1, deriving this explicit analytical solution for a flux under a fixed force constraint is equivalent to computing the derivative of the corresponding Legendre-Fenchel dynamic free energy potential.

2. The Positivity of J_{leak}: The requirement that the compensatory leak flux is strictly positive ($J_{\text{leak}} > 0$) is a direct consequence of probability conservation combined with the thermodynamic constraint of the futile loop ($\mathfrak{F}_{\text{leak}} = 0$). Because the catalytic step is strictly non-invertible ($k_{\text{cat}} > 0$), it acts as a constant sink, driving a unidirectional probability flux $p_{\text{cat}} = \pi_1 k_{\text{cat}} > 0$ from node 1 (ES) to node 0 (E). To maintain a steady state, node 1 must continuously receive a net incoming probability flux from its connected pathways to replenish this loss. Kirchhoff's current law thus requires $J_{01} + J_{21} = p_{\text{cat}} > 0$. Since the unbranched sideway route enforces $J_{21} = J_{\text{leak}}$, we obtain the flux conservation condition:

$$J_{01} + J_{\text{leak}} > 0. \tag{S78}$$

This physical boundary dictates that the sum of the primary and leak fluxes must be strictly positive.

Furthermore, the flows J_{01} and J_{leak} are bound by the zero cycle force constraint along the inhibitor loop. As derived in Eq. (S76), the affinity of the primary edge must perfectly balance the total affinity of the sideway edges:

$$\text{arctanh}\left(\frac{J_{01}}{\tau_{01}}\right) = \text{arctanh}\left(\frac{J_{\text{leak}}}{\tau_{12}}\right) + \text{arctanh}\left(\frac{J_{\text{leak}}}{\tau_{23}}\right) + \text{arctanh}\left(\frac{J_{\text{leak}}}{\tau_{30}}\right). \tag{S79}$$

The function $\text{arctanh}(x)$ is strictly monotonically increasing and odd, which means it preserves the sign of its argument. Therefore, the total sum on the right-hand side shares the exact same sign as J_{leak}. For the thermodynamic equality to hold, J_{01} and J_{leak} must strictly share the same sign (or both be zero).

Since J_{01} and J_{leak} must have the same sign, and their sum must be strictly positive to replenish the catalytic sink ($J_{01} + J_{\text{leak}} > 0$), they cannot be zero or negative. Both fluxes must therefore be strictly positive. This guarantees that the noncompetitive inhibitor loop inevitably acts as a forward compensatory shunt ($J_{\text{leak}} > 0$).

3. Proof of Monotonic Coupling via Chain Rule: We evaluate the sensitivity of production to the leak, $\partial J_{\text{cat}}/\partial J_{\text{leak}}$, within the mixed coordinate $\mathbf{z} = (\boldsymbol{\pi}, \boldsymbol{\tau}, J_{\text{leak}}, \mathfrak{F}_{\text{leak}})$. This dictates that the partial derivative is taken while holding the kinetic traffic $\boldsymbol{\tau}$ and the cycle force $\mathfrak{F}_{\text{leak}} = 0$ constant. Using the derivative rules $\frac{d}{dx}\tanh(u) = \text{sech}^2(u)\frac{du}{dx}$ and $\frac{d}{dx}\text{arctanh}(x/a) = \frac{a}{a^2-x^2}$, the derivative of the sideway affinity evaluates to:

$$\frac{\partial \mathcal{A}_{\text{side}}}{\partial J_{\text{leak}}} = \sum_{k\in\text{side}} \frac{\tau_k}{\tau_k^2 - J_{\text{leak}}^2}. \tag{S80}$$

Thus, the total derivative of the catalytic flux is:

$$\frac{\partial J_{\text{cat}}}{\partial J_{\text{leak}}} = 1 + \tau_{01}\underbrace{\text{sech}^2(\mathcal{A}_{\text{side}})}_{>0}\underbrace{\left(\sum_{k\in\text{side}} \frac{\tau_k}{\tau_k^2 - J_{\text{leak}}^2}\right)}_{>0}. \tag{S81}$$

Since all edge traffics bound their net fluxes ($\tau_k > |J_{\text{leak}}|$) for any physical NESS, the derivative $\partial J_{\text{cat}}/\partial J_{\text{leak}}$ is greater than 1. This proves that J_{cat} and J_{leak} are locked in a positive coupling.

4. Jensen's Inequality and Global Bounds on Production: To identify the limits of inhibitor efficacy, we analyze how the distribution of sideway traffic τ_k modulates the production flux J_{cat} under a fixed total traffic budget $\sum \tau_k = 3\bar{\tau}$.

The resistance function $f(\tau) = \text{arctanh}(J_{\text{leak}}/\tau)$ is convex ($f''(\tau) > 0$) for all physical regimes where $\tau > J_{\text{leak}}$. This convexity leads to two bounds:

(A) The Lower Bound (J_{cat}^{min}) — The Inhibitor Equal Traffic Principle: By Jensen's Inequality, the sum of a convex function is minimized when all input variables are perfectly equal:

$$\mathcal{A}_{\text{side}} = \sum_{k=1}^{3} \text{arctanh}\left(\frac{J_{\text{leak}}}{\tau_k}\right) \geq 3\text{arctanh}\left(\frac{J_{\text{leak}}}{\bar{\tau}}\right). \tag{S82}$$

Substituting this minimum affinity into the flux-coupling formula Eq. (S77), we obtain the minimum for the production flux:

$$J_{\text{cat}}^{\text{min}} = J_{\text{leak}} + \tau_{01} \tanh\left(3\text{arctanh}\frac{J_{\text{leak}}}{\bar{\tau}}\right). \tag{S83}$$

This confirms that the inhibitor achieves its maximal efficacy (minimal J_{cat}) when the kinetic traffic is balanced across the entire inhibitor cycle.

(B) The Upper Bound (J_{cat}^{max}) — The Bottleneck Limit: Conversely, the maximum of a convex function under a sum constraint occurs at the boundaries of the feasible domain. For J_{cat} to be maximized, the sideway traffic distribution must be maximally uneven.

Consider the limit where one transition in the inhibitor cycle, say τ_1, becomes a kinetic bottleneck such that $\tau_1 \to J_{\text{leak}}^+$. In this limit:

1. The local resistance of that edge diverges: $\text{arctanh}(J_{\text{leak}}/\tau_1) \to \infty$.
2. Consequently, the total sideway resistance diverges: $\mathcal{A}_{\text{side}} \to \infty$.
3. Using the asymptotic property of the hyperbolic tangent, $\lim_{x\to\infty} \tanh(x) = 1$.

Substituting this limit into Eq. (S77), we derive the upper bound for production:

$$J_{\text{cat}}^{\text{max}} = J_{\text{leak}} + \tau_{01}. \tag{S84}$$

This upper bound reflects the state of the inhibitor loop: a bottleneck severs the compensatory leak, freeing the production flux to its maximum capacity dictated by the primary binding traffic τ_{01}.

We numerically validate these lower and upper bounds of J_{cat} in the main text by log-uniformly sampling 10^5 networks with transition rates spanning six orders of magnitude (from 10^{-3} to 10^3) under the futile loop constraint.
